# Novel Caryophyllane-Related Sesquiterpenoids with Anti-Inflammatory Activity from *Rumphella antipathes* (Linnaeus, 1758)

**DOI:** 10.3390/md18110554

**Published:** 2020-11-06

**Authors:** Yu-Chia Chang, Chih-Chao Chiang, Yuan-Shiun Chang, Jih-Jung Chen, Wei-Hsien Wang, Lee-Shing Fang, Hsu-Ming Chung, Tsong-Long Hwang, Ping-Jyun Sung

**Affiliations:** 1 Research Center for Chinese Herbal Medicine, Graduate Institute of Healthy Industry Technology, College of Human Ecology, Chang Gung University of Science and Technology, Taoyuan 333324, Taiwan; ycchang03@mail.cgust.edu.tw; 2 Graduate Institute of Clinical Medical Sciences, College of Medicine, Chang Gung University, Taoyuan 333323, Taiwan; D0600501@cgu.edu.tw; 3 Department of Chinese Pharmaceutical Sciences and Chinese Medicine Resources, College of Chinese Medicine, China Medical University, Taichung 404333, Taiwan; yschang@mail.cmu.edu.tw; 4 Faculty of Pharmacy, School of Pharmaceutical Sciences, National Yang-Ming University, Taipei 112304, Taiwan; chenjj@ym.edu.tw; 5 Department of Marine Biotechnology and Resources, College of Marine Sciences, National Sun Yat-sen University, Kaohsiung 804201, Taiwan; whw@mail.nsysu.edu.tw (W.-H.W.); lsfang@gcloud.csu.edu.tw (L.-S.F.); 6 Center for Environmental Toxin and Emerging-Contaminant Research, Cheng Shiu University, Kaohsiung 833301, Taiwan; 7 Super Micro Mass Research and Technology Center, Cheng Shiu University, Kaohsiung 833301, Taiwan; 8 Department of Applied Chemistry, College of Science, National Pingtung University, Pingtung 900393, Taiwan; 9 Graduate Institute of Natural Products, College of Medicine, Chang Gung University, Taoyuan 333323, Taiwan; 10 Department of Anesthesiology, Chang Gung Memorial Hospital, Taoyuan 333423, Taiwan; 11 Department of Chemical Engineering, College of Environment and Resources, Ming Chi University of Technology, New Taipei City 243303, Taiwan; 12 Research Center for Food and Cosmetic Safety, College of Human Ecology, Chang Gung University of Science and Technology, Taoyuan 333324, Taiwan; 13 National Museum of Marine Biology and Aquarium, Pingtung 944401, Taiwan; 14 Graduate Institute of Marine Biology, College of Marine Sciences, National Dong Hwa University, Pingtung 944401, Taiwan; 15 Chinese Medicine Research and Development Center, China Medical University Hospital, Taichung 404394, Taiwan; 16 Graduate Institute of Natural Products, College of Pharmacy, Kaohsiung Medical University, Kaohsiung 807378, Taiwan

**Keywords:** *Rumphella antipathes*, antipacid, caryophyllane, clovane, rumphellolide, superoxide anion, elastase

## Abstract

Two previously undescribed caryophyllane-related sesquiterpenoids, antipacids A (**1**) and B (**2**), with a novel bicyclo[5.2.0] core skeleton, and known compound clovane-2β,9α-diol (**3**), along with rumphellolide L (**4**), an esterified product of **1** and **3**, were isolated from the organic extract of octocoral *Rumphella antipathes*. Their structures, including the absolute configurations were elucidated by spectroscopic and chemical experiments. In vivo anti-inflammatory activity analysis indicated that antipacid B (**2**) inhibited the generation of superoxide anions and the release of elastase by human neutrophils, with IC_50_ values of 11.22 and 23.53 μM, respectively, while rumphellolide L (**4**) suppressed the release of elastase with an IC_50_ value of 7.63 μM.

## 1. Introduction

**Figure 1:**
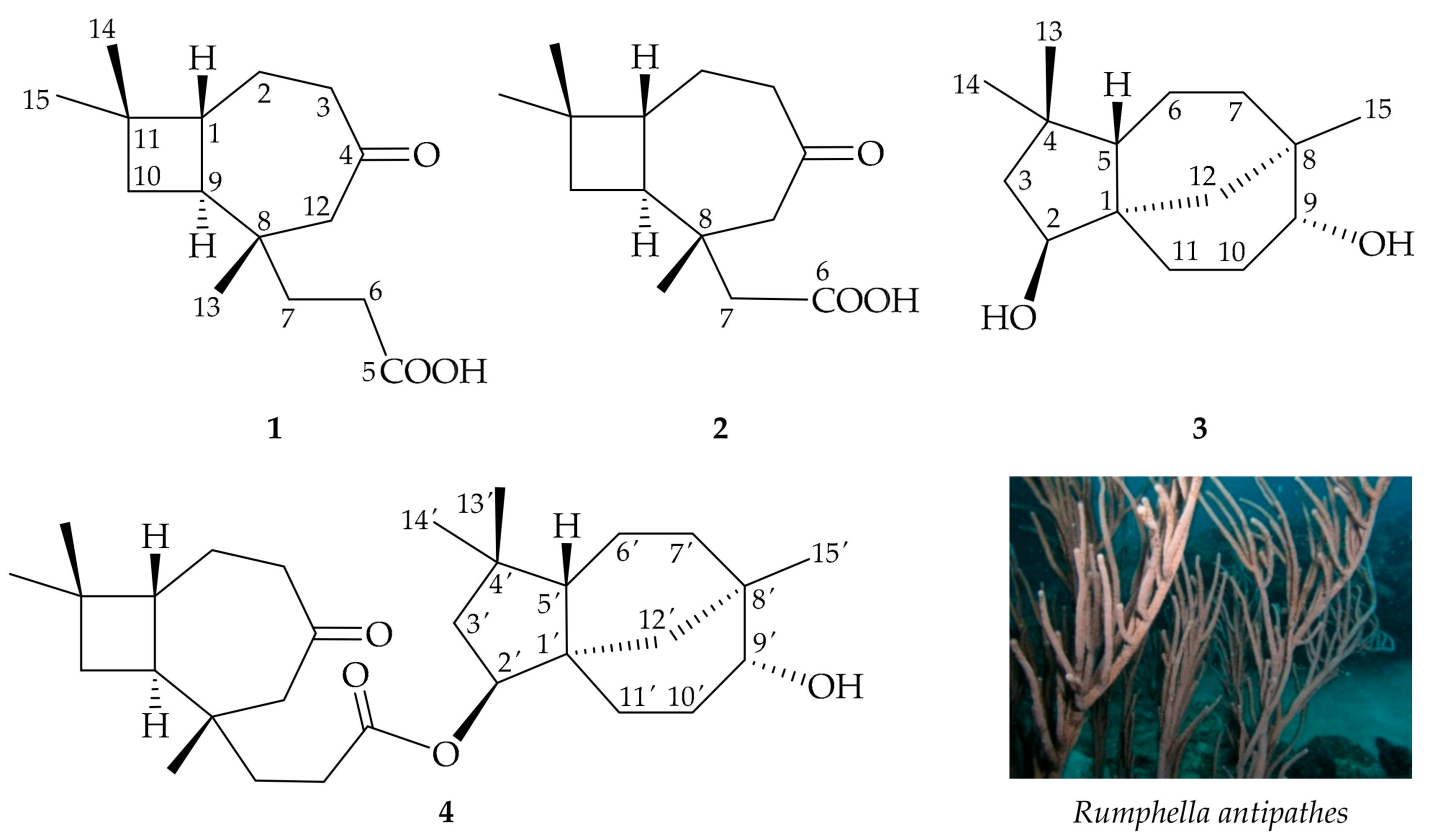
Structures of antipacids A (**1**) and B (**2**), clovane-2β,9α-diol (**3**), and rumphellolide L (**4**), and an image of *Rumphella antipathes*.

*Rumphella* (family Gorgoniidae) is a genus of soft coral consisting of four species, *R. aggregata*, *R. antipathes*, *R. suffruticosa*, and *R. torta*, the center of marine diversity of this genus being found in the Indo-Pacific Ocean. Corals were described by Shi-Zhen Li in his ancient herbal *Compendium of Chinese Materia Medica*, published in 1596, as “sweet, neutral and non-toxic; used to remove eye vision obstruction; clear abiding static blood; blow the powder to nose to stop nose bleeding; brighten the eye and calm the spirit; stop epileptic seizure; apply to the eye to improve floater.” Previous studies showed that the *Rumphella* genus exhibited extensive bioactivities, including antiproliferative [1], cytotoxic [2,3,4], antifungal [5], antibacterial [6,7,8,9], and anti-inflammatory [10,11,12,13,14,15,16,17,18] activities. Studies of the chemical constituents of octocorals of the *Rumphella* genus have led to the isolation of a series of compounds, including caryophyllanes [2,6,7,8,9,10,11,12,16,17,18,19,20,21,22], clovanes [13,14,15,23], steroids [3,4,5,24,25], glycerols [5], and fatty acids and lipids [25,26,27,28,29]. Our continuing studies of the constituents of the same extract from *R. antipathes* (Figure 1) resulted in the isolation of two novel caryophyllane-related sesquiterpenoids, antipacids A (**1**) and B (**2**), featuring a bicyclo[5.2.0] carbon core; a known sesquiterpenoid, clovane-2β,9α-diol (**3**); and rumphellolide L (**4**), an esterified product of **1** and **3** (Figure 1). This paper describes the isolation, structure determination, biosynthetic pathway analysis, and anti-inflammatory properties of sesquiterpenoids **1**–**4**.

## 2. Results and Discussion

**Figure 2:**
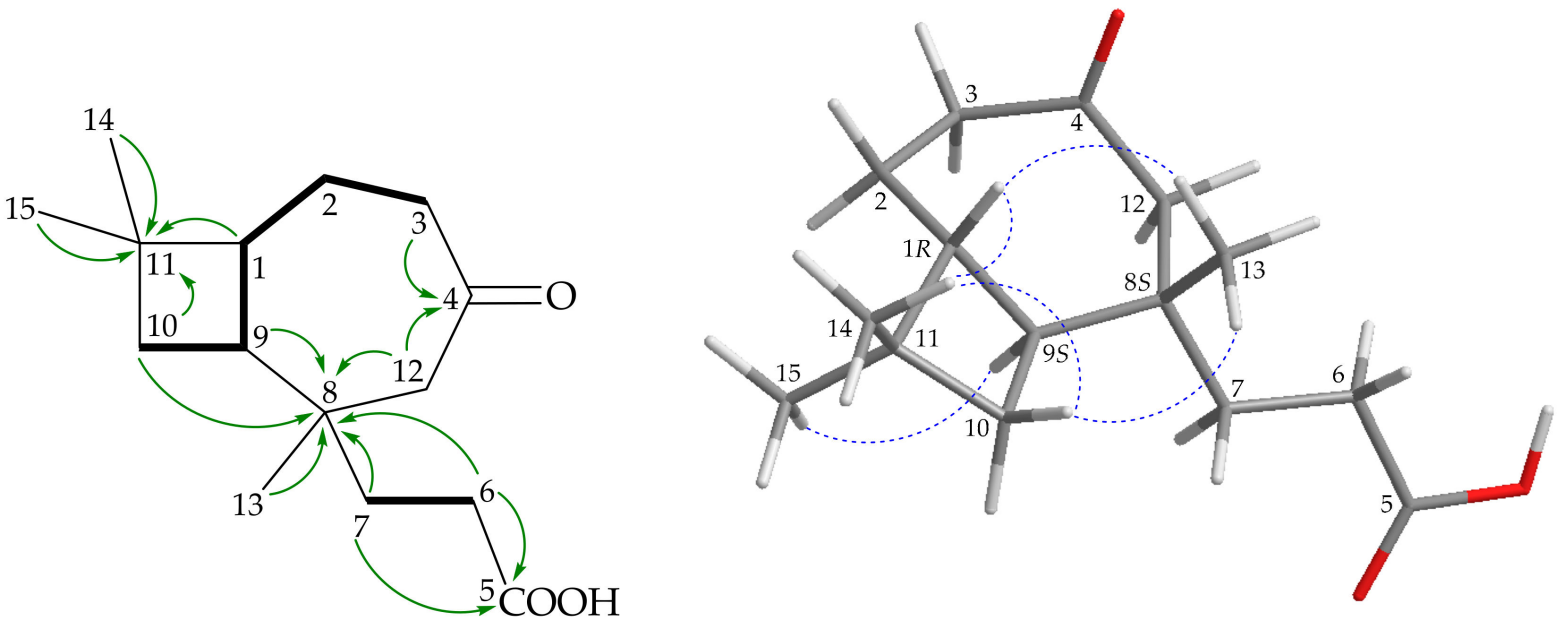
(**A**) Key COSY (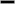), HMBC (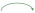), and (**B**) NOESY (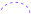) correlations of **1**.

Antipacid A (**1**) was obtained as a colorless colloid, showing an electrospray ionization mass spectrum (ESIMS) quasimolecular ion peak at *m/z* 253, and was found to have the molecular formula C_15_H_24_O_3_ by analysis of ^13^C and ^1^H NMR data (Table 1); this conclusion was confirmed by a positive- mode high-resolution-ESIMS ([+]-HRESIMS) peak at *m/z* 253.1792 [M + H]^+^ (calcd. for C_15_H_24_O_3_ + H, 253.1789), with four indexes of hydrogen deficiency. The IR spectrum showed absorption bands at 3600–2400 (carboxyl group) and 1708 cm^−1^ (ketonic carbonyl). From the ^13^C NMR data of **1** (Table 1), ketonic (δ_C_ 212.8, C-4) and carboxyl (δ_C_ 179.1, C-5) groups were deemed present. Thus, **1** was identified as a bicyclic compound. ^1^H–^1^H correlation spectroscopy (COSY) enabled identification of two spin systems, H_2_-10/H-9/H-1/H_2_-2/H_2_-3 and H_2_-6/H_2_-7 (Figure 2). These findings, together with the ^2^*J*- and ^3^*J*-^1^H–^13^C long-range correlations between protons and non-protonated carbons, such as H_2_-3, H_2_-12/C-4; H_2_-6, H_2_-7/C-5; H_2_-6, H_2_-7, H-9, H_2_-10, H_2_-12, H_3_-13/C-8; and H-1, H_2_-10, H_3_-14, H_3_-15/C-11 in the heteronuclear multiple-bond coherence (HMBC) experiment (Figure 2), permitted elucidation of the main carbon skeleton of **1**.

The relative configuration of **1** was assigned from the results of a nuclear Overhauser effect spectroscopy (NOESY) experiment (Figure 2) and vicinal coupling constants. The *trans* geometries of H-9 (δ_H_ 1.87) and H-1 (δ_H_ 1.77) were indicated by a large coupling constant (*J* = 10.4 Hz) between these two ring juncture protons, and H-9 and H-1 were α- and β-oriented, respectively. H-1 exhibited a correlation with H_3_-13, setting Me-13 at C-8 on the β face. Based on the above findings, the stereogenic carbons of **1** were elucidated as (1R*,8S*,9S*). Antipacids A (**1**) and B (**2**) were isolated along with natural products rumphellaone A, a novel 4,5-*seco*-caryophyllane [2], and (8*R*,9*R*)-isocaryolane-8,9-diol [21,30] (the numbering system used in reference [30] was different to that in this study) from the same target organism, *R. antipathes* [2,21]. The structures, including the absolute configurations, of rumphellaone A [31,32,33] and (8*R*,9*R*)-isocaryolane-8,9-diol [30], were confirmed by synthetic methods. Based on these findings and previous studies [2,6,7,8,9,10,11,12,16,17,18,19,20,21,22], all marine-origin naturally occurring caryophyllane-type sesquiterpenoids have the H-9 *trans* to H-1, which are assigned as α- and β-oriented, respectively. Therefore, it is reasonable on biogenetic grounds to suggest that **1** and **2** have the same absolute configuration as rumphellaone A and (8*R*,9*R*)-isocaryolane-8,9-diol, tentatively, and the configurations of the stereogenic carbons of **1** can be elucidated as (1*R*,8*S*,9*S*) (Supplementary Materials, Figures S1–S7).

**Figure 3:**
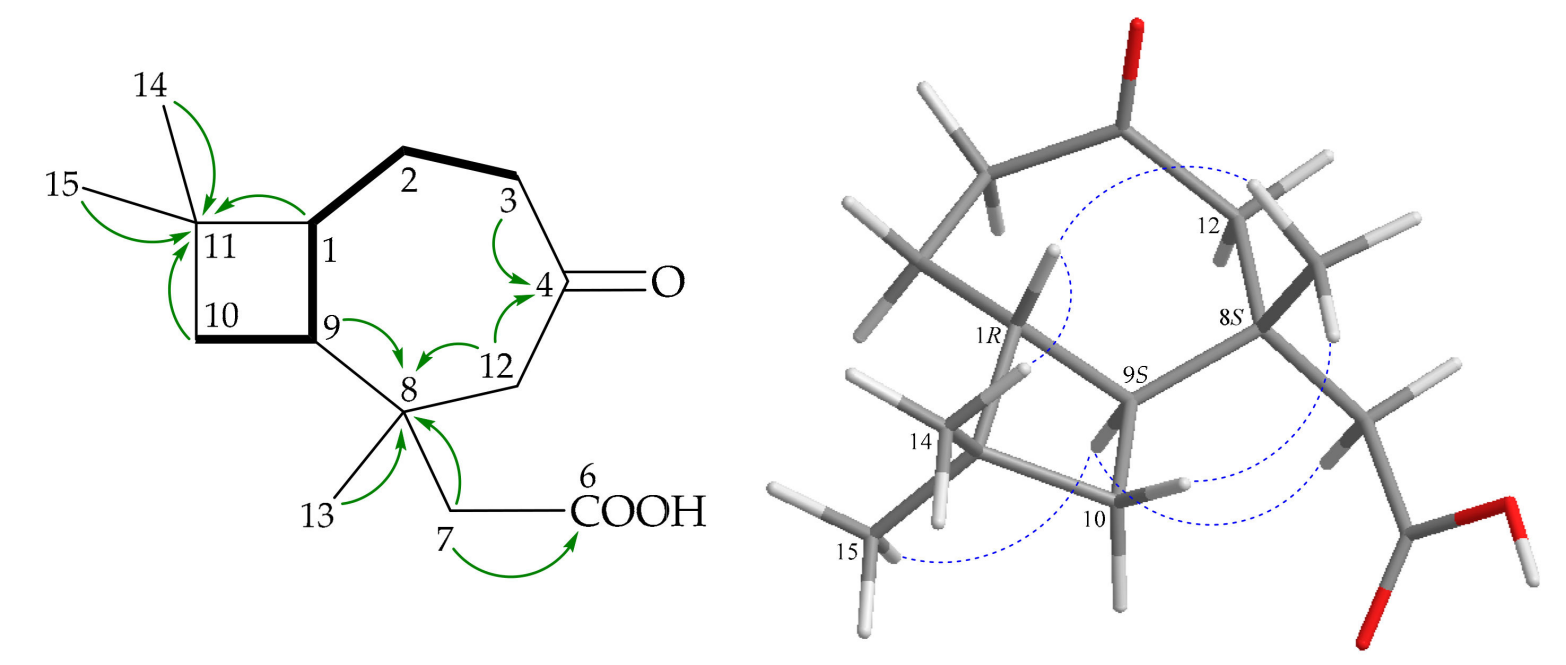
Key COSY (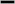), HMBC (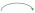), and NOESY (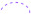) correlations of **2**.

Antipacid B (**2**) was isolated as a colorless colloid that showed a sodiated adduct ion peak in (+)-HRESIMS at *m/z* 261.1468 [M + Na]^+^, which accounted for the molecular formula, C_14_H_22_O_3_ (calcd. for C_14_H_22_O_3_ + Na, 261.1467), with 4 degrees of unsaturation. The spectroscopic data of **2** resembled those of **1** (Table 1). The one-dimensional (1D) and two-dimensional (2D) NMR spectra revealed that the signals corresponding to the propanoic acid moiety in **1** were replaced by those of an acetic acid in **2** (Figure 3). Therefore, **2** was assigned as having a structure with the same stereochemistry as **1** because of the stereogenic carbons that **2** had in common with **1** by correlations observed in the NOESY spectrum (Figure 3); therefore, the configurations of the stereogenic carbons of **2** were elucidated as (1*R*,8*S*,9*S*) (Supplementary Materials, Figures S8–S14). 

**Figure 4:**
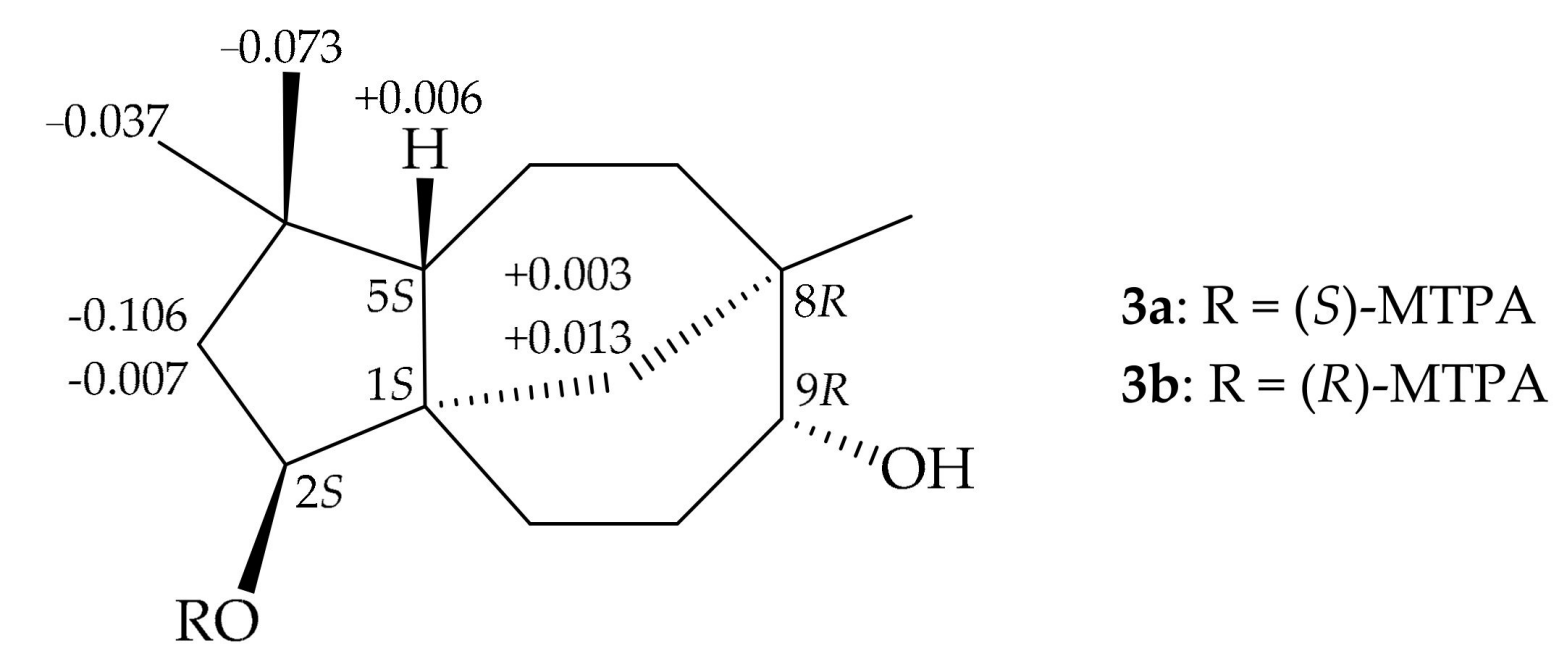
^1^H NMR chemical shift differences Δδ (δ_S_–δ_R_) in ppm for the MPTA esters of **3**.

Compound **3** was identified by comparison of its spectroscopic data with those of clovane-2β,9α-diol, which had been previously isolated from terrestrial plants *Dipterocarpus pilosus* [34], *Salvia canariensis* [35], *Viguiera* *excelsa* [36], *Viguiera* *linearis* [37], and *Sindora sumatrana* [38]. This was the first occasion in which this metabolite was obtained from a marine source. Clovane **3** was treated with (*R*)-(–)- and (*S*)-(+)-MTPA chloride to yield (*S*)- and (*R*)-MTPA esters **3a** and **3b**, respectively. A comparison of the ^1^H NMR chemical shifts of **3a** and **3b** (Δδ values shown in Figure 4) led to the assignment of the *S*-configuration at C-2 (Supplementary Materials, Figures S15–S16). Therefore, the absolute configurations of the stereogenic centers of **3** were determined as (1*S*,2*S*,5*S*,8*R*,9*R*).

Rumphellolide L (**4**) was isolated as a colorless colloid that showed a sodiated adduct ion peak [M + Na]^+^ at *m/z* 495.3447 in (+)-HRESIMS. The result revealed that this compound had a molecular formula of C_30_H_48_O_4_ (calcd. for C_30_H_48_O_4_ + Na, 495.3450), with 7 degrees of unsaturation. Strong bands at 3485, 1731, and 1704 cm^−1^ in the IR spectrum indicated the presence of hydroxy, ester, and ketonic groups. The ^13^C NMR and distortionless enhancement by polarization transfer (DEPT) spectra revealed that **4** had 30 carbons (Table 2), including six methyls, twelve methylenes, five methines (including two oxymethines), five sp^3^ quaternary carbons, an ester carbonyl, and a ketonic carbonyl. Therefore, **4** was identified as having five rings.

**Figure 5:**
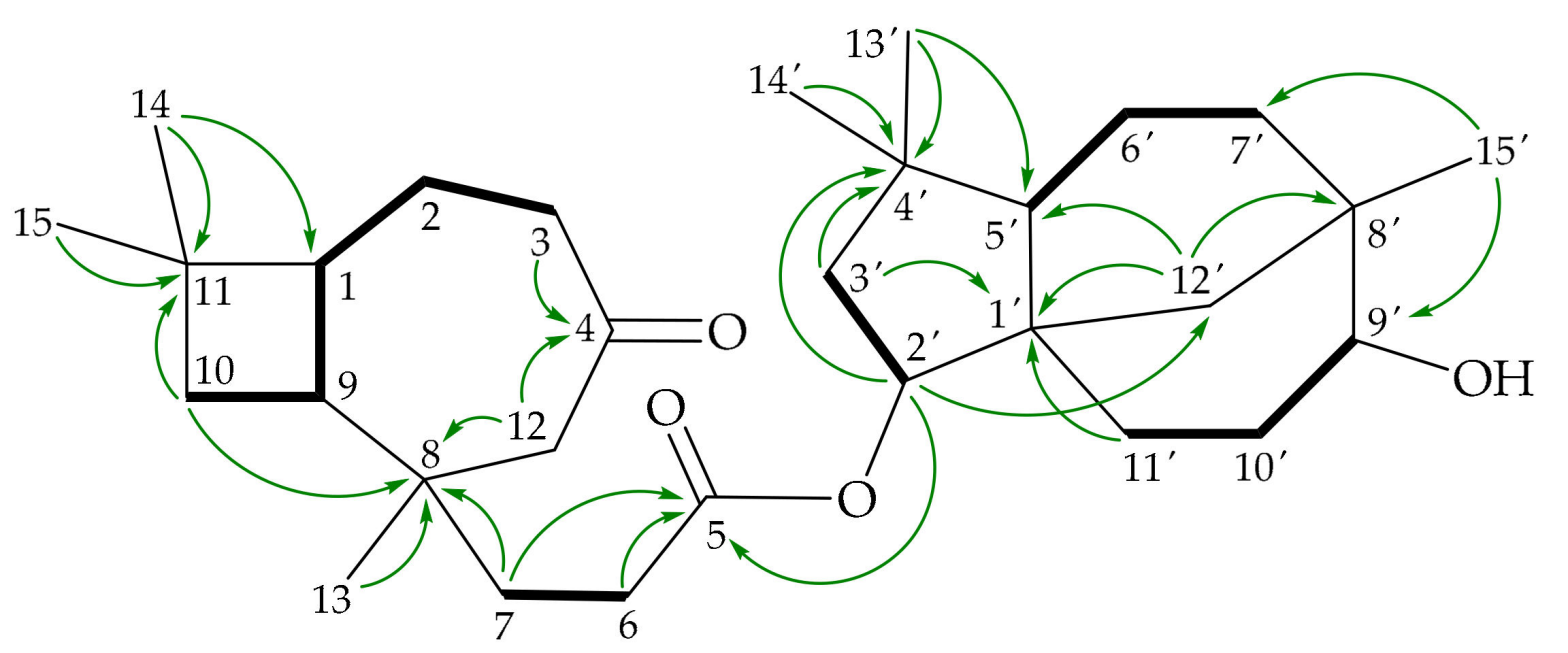
Key COSY (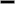) and HMBC (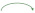) correlations of **4**.

From the ^1^H–^1^H COSY spectrum, the data differentiated the spin systems H_2_-10/H-9/H-1/H_2_-2/H_2_-3, H_2_-6/H_2_-7, H-2’/H_2_-3´, H-5´/H_2_-6´/H_2_-7´, and H-9´/H_2_-10´/H_2_-11´ (Figure 5), and these findings together with the results of key HMBC correlations shown in Figure 5 confirmed the carbon skeleton of **4**. An HMBC correlation between H-2´ (δ_H_ 4.83), an oxymethine proton, and the C-5 ester carbonyl carbon (δ_C_ 173.6) was found, which proved the existence of an ester linkage in **4**. It was found that the NMR data were similar to those of **1** and **3**, and this compound was proven to be the dehydrated product of **1** and **3**. Due to the absolute configurations of **1** and **3** having been determined, the absolute configurations of the stereogenic carbons of **4** were assigned as (1*R*,8*S*,9*S*,1´*S*,2´*S*,5´*S*,8´*R*,9´*R*) (Supplementary Materials, Figures S17–S23).

**Scheme 1:**
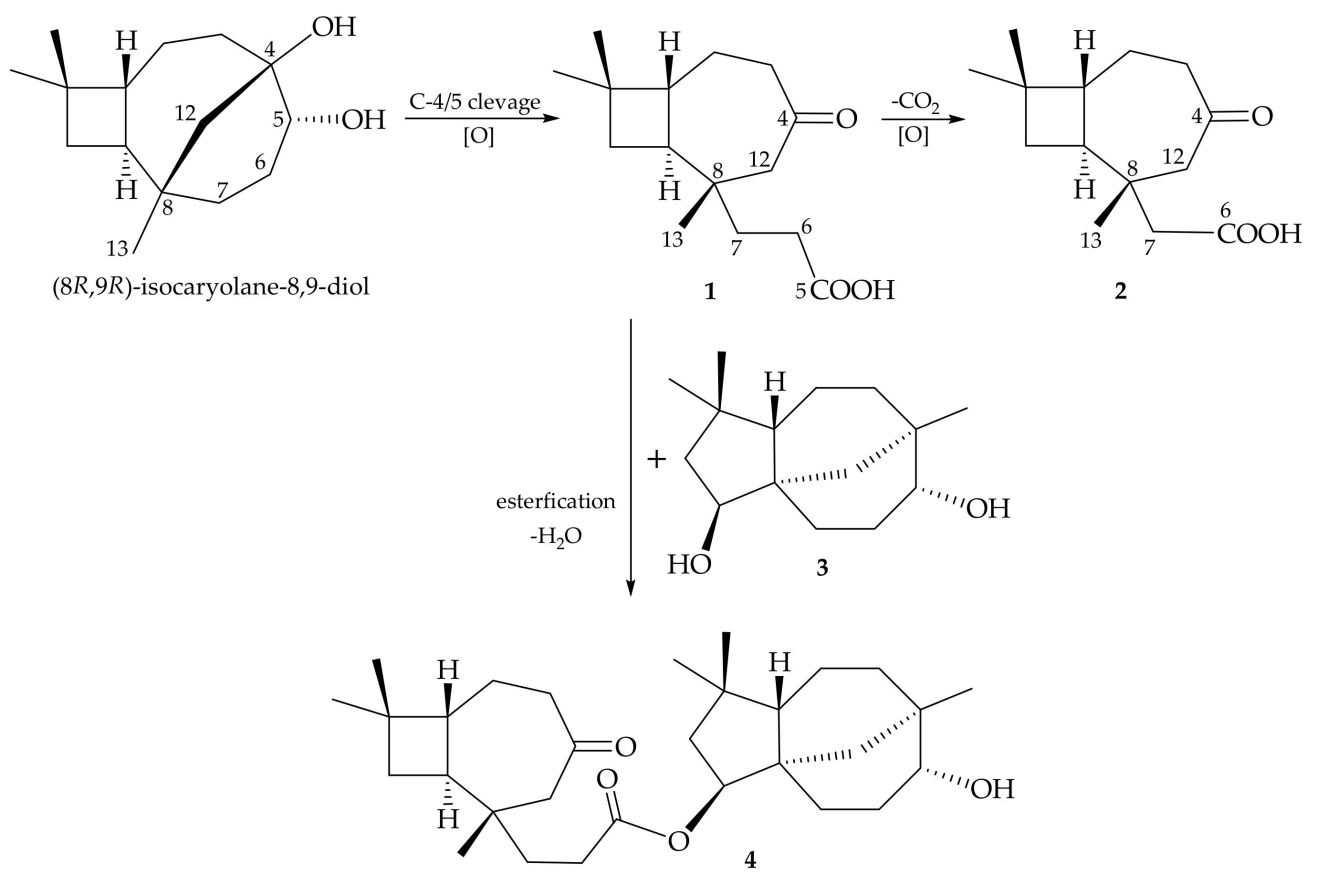
Plausible biogenetic pathway of **1**–**4**.

The proposed biogenetic pathway of sesquiterpenoids **1**–**4** is outlined in Scheme 1. The ring- opening reaction might be rationally derived from (8*R*,9*R*)-isocaryolane-8,9-diol [30] (the numbering system used in reference [30] was different to that in this study), which had also been isolated from *R. antipathes* [21], and might subsequently, under oxidation, produce the carbon skeletons of **1** and **2**.

The in vitro anti-inflammatory effects of **1**–**4** were assessed (Table 3). Antipacid B (**2**) displayed inhibitory effects on the generation of superoxide anions and the release of elastase by human neutrophils (IC_50_ = 11.22 and 23.53 μM, respectively). Antipacid A (**1**) did not show activity, implying that the presence of a large substituent at C-8 weakens the activity in comparison with the structure and anti-inflammatory activities of **2**. Although **1** and **3** were not active, rumphellolide L (**4**), the dehydrated product of **1** and **3** with esterification, showed activity in inhibiting the release of elastase (IC_50_ = 7.63 μM).

## 3. Materials and Methods

### 3.1. General Experimental Procedures

Optical rotations were recorded on a JASCO-P1010 polarimeter (Japan Spectroscopic Corporation, Tokyo, Japan). IR spectra were obtained on a Varian Diglab FTS 1000 FT-IR spectrometer (Varian Inc., Palo Alto, CA, USA). NMR spectra were recorded on a Varian Mercury Plus 400 spectrometer (400 MHz for ^1^H and 100 MHz for ^13^C) (Varian Inc.) using the residual CHCl_3_ (δ_H_ 7.26 ppm) and CDCl_3_ (δ_C_ 77.1 ppm) signals as internal references for ^1^H and ^13^C NMR, respectively.

Chemical shifts are shown in δ (ppm) and coupling constants (*J*) are given in Hz. ESIMS and HRESIMS data were recorded using a Bruker APEX II FTMS system (Bremen, Germany). Silica gel (230–400 mesh, Merck, Darmstadt, Germany) was used for column chromatography. Thin-layer chromatography (TLC) was performed on plates precoated with Kieselgel 60 F_254_ (0.25-mm-thick, Merck), then sprayed with 10% H_2_SO_4_ solution followed by heating to visualize the spots. Normal-phase HPLC (NP-HPLC) (Hitachi L-7100 series using a L-7455 photodiode array detector, Hitachi Ltd., Tokyo, Japan; and a semi-preparative Hibar 250 mm × 10 mm, LiChrospher Si 60, 5 μm column, Merck) was employed.

### 3.2. Animal Material

The octocoral *R. antipathes* (Linnaeus, 1758) was collected by hand by self-contained underwater breathing apparatus (SCUBA) divers off the coast of South Taiwan in May 2004. The samples were stored in a −20 °C freezer until used for extraction. Identification of the species of this organism was performed by comparison as described in previous studies [39,40]. A voucher specimen (no.: NMMBA-TWGC-010) was deposited in the National Museum of Marine Biology and Aquarium, Taiwan.

### 3.3. Extraction and Isolation

*R. antipathes* (wet/dry weight = 402/144 g) was sliced and then extracted with a solvent mixture of MeOH and dichloromethane (DCM) (1:1). The extract was partitioned between ethyl acetate (EtOAc) and H_2_O. The EtOAc layer (1.23 g) was then applied on silica gel column and eluted with gradients of hexanes/EtOAc (from 25:1 to 100% EtOAc) to furnish 29 subfractions. Fraction 18 was purified by NP-HPLC using a solvent mixture of *n*-hexane/EtOAc (5:1; at a flow rate = 3.0 mL/min) to yield **4** (3.5 mg, 5:1). Fraction 22 was separated by NP-HPLC using a mixture of DCM and EtOAc (10:1; at a flow rate = 5.0 mL/min) to afford **2** (3.5 mg). Fraction 24 was separated by NP-HPLC using a mixture of *n*-hexane and EtOAc (1:1; at a flow rate = 5.0 mL/min) to afford **1** (5.8 mg) and **3** (60.1 mg), respectively.

Antipacid A (**1**): Colorless colloid; [α]25D −9.2 (*c* 0.29, CHCl_3_); IR (neat) ν_max_ 3600−2400 (broad), 1708 cm^−1^; ^1^H and ^13^C NMR data, see Table 1; ESIMS: *m/z* 253 [M + H]^+^; HRESIMS: *m/z* 253.1792 [M + H]^+^ (calcd. for C_15_H_24_O_3_ + H, 253.1789).

Antipacid B (**2**): Colorless colloid; [α]25D −9.4 (*c* 0.18, CHCl_3_); IR (neat) ν_max_ 3600−2600 (broad), 1710 cm^−1^; ^1^H and ^13^C NMR data, see Table 1; ESIMS: *m/z* 261 [M + Na]^+^; HRESIMS: *m/z* 261.1468 [M + Na]^+^ (calcd. for C_14_H_22_O_3_ + Na, 261.1467).

Clovane-2β,9α-diol (**3**): Amorphous powder; [α]23D +3.5 (*c* 1.82, CHCl_3_) (ref. [38] [α] D +3.19 (*c* 2.27, CHCl_3_)); IR (neat) ν_max_ 3378 cm^−1^; ^1^H (400 MHz, CDCl_3_) and ^13^C (100 MHz, CDCl_3_) NMR data were found to be in complete agreement with a previous report [37]; ESIMS: *m/z* 261 [M + Na]^+^.

Rumphellolide L (**4**): Colorless colloid; [α]25D −7.5 (*c* 0.18, CHCl_3_); IR (neat) ν_max_ 3485, 1731, 1704 cm^−1^; ^1^H and ^13^C NMR data, see Table 2; ESIMS: *m/z* 495 [M + Na]^+^; HRESIMS: *m/z* 495.3447 [M + Na]^+^ (calcd. for C_30_H_48_O_4_ + Na, 495.3450).

### 3.4. (S)- and (R)-MTPA Esters of 3

To a solution of **3** (10.0 mg) in pyridine (0.4 mL) (−)-α-methoxy-α-(trifluoromethyl)-phenylacetyl (MTPA) chloride was added (25.0 μL) at 25 °C for 4−5 h. The mixture was dried and purified by a silica gel column with *n*-hexane/EtOAc (10:1) to give (*S*)-MTPA ester **3a** (8.5 mg). The (*R*)-MTPA ester **3b** (0.2 mg) was prepared from (+)-MTPA chloride by the same method (10 mg compound **3** was used). Selected Δδ values are shown in Figure 4. 

### 3.5. Superoxide Anion Generation and Elastase Release by Human Neutrophils

The proinflammatory suppression assay was employed to assess the activities of isolated compounds **1**–**4** against the generation of superoxide anions and the release of elastase by human neutrophils according to the protocols described in the literature [41].

## 4. Conclusions

The current work illustrated the anti-neutrophilic inflammatory properties of caryophyllane-related sesquiterpenoids, and two metabolites with novel structures, antipacids A and B (**1** and **2**), clovane-2β,9α-diol (**3**), and rumphellolide L (**4**), an esterified product of **1** and **3**, were isolated from *R. antipathes*. Compound **2** displayed inhibitory effects on the generation of superoxide anions and the release of elastase, and **4** showed activity in suppressing the release of elastase. These results indicated a structural-dependent specificity of C-8 in **1**, **2**, and **4** in neutrophilic targets, which will motivate future research examining this specificity, as well as clarify the molecular mechanisms of the active leads.

## Figures and Tables

**Table 1:** ^1^H (400 MHz, CDCl_3_) and ^13^C (100 MHz, CDCl_3_) NMR data of **1** and **2**.

	1		2	
C/H	δ_H_ (*J* in Hz)	δ_C_, Type	δ_H_ (*J* in Hz)	δ_C_, Type
1	1.77 ddd (10.4, 10.4, 3.6)	45.3, CH	1.81 ddd (10.8, 10.8, 3.6)	44.9, CH
2a/b	1.70 m; 1.64 m	23.7, CH_2_	1.71 m; 1.62 m	23.7, CH_2_
3a/b	2.48 m; 2.41 m	43.8, CH_2_	2.49 m	43.7, CH_2_
4	-	212.8, C	-	212.5, C
5	-	179.1, C	-	-
6	2.27 m	29.3, CH_2_	-	176.0, C
7a/b	1.72 m; 1.53 m	36.5, CH_2_	2.29 d (13.6); 2.28 d (13.6)	44.7, CH_2_
8	-	35.0, C	-	34.9, C
9	1.87 ddd (10.4, 10.4, 8.0)	46.3, CH	1.98 ddd (10.8, 10.8, 8.4)	46.2, CH
10a/b	1.57 dd (10.4, 8.0); 1.49 dd (10.4, 10.4)	35.5, CH_2_	1.56 dd (10.8, 8.4); 1.48 dd (10.8, 10.8)	34.9, CH_2_
11	-	34.4, C	-	33.9, C
12a/b	2.35 d (11.2); 2.30 d (11.2)	54.8, CH_2_	2.60 d (11.2); 2.49 d (11.2)	54.1, CH_2_
13	0.92 s	20.5, CH_3_	1.08 s	21.2, CH_3_
14	1.01 s	30.1, CH_3_	1.02 s	30.1, CH_3_
15	1.01 s	22.1, CH_3_	1.01 s	22.1, CH_3_

**Table 2:** ^1^H (400 MHz, CDCl_3_) and ^13^C (100 MHz, CDCl_3_) NMR data for **4**.

C/H	δ_H_ (*J* in Hz)	δ_C_, Type	C/H	δ_H_ (*J* in Hz)	δ_C_, Type
1	1.77 m	45.2, CH	1´	-	44.5, C
2	1.64 m	23.6, CH_2_	2´	4.83 dd (8.8, 6.0)	82.1, CH
3a/b	2.48 ddd (12.4, 7.6, 4.0); 2.39 m	43.7, CH_2_	3´a/b	1.78 dd (12.0, 6.0); 1.51 m	44.3, CH_2_
4	-	212.2, C	4´	-	38.0, C
5	-	173.6, C	5´	1.48 m	50.3, CH
6	2.21 t (7.6)	29.8, CH_2_	6´	1.46 m	20.8, CH_2_
7	1.73 m	36.7, CH_2_	7´	1.40 m	33.0, CH_2_
8	-	35.1, C	8´	-	34.6, C
9	1.87 ddd (10.8, 10.8, 8.4)	46.4, CH	9´	3.31 br s	74.9, CH
10	1.54 m	35.5, CH_2_	10´a/b	2.00 m; 1.65 m	26.3, CH_2_
11	-	34.4, C	11´	1.58 m	27.3, CH_2_
12	2.31 s	54.9, CH_2_	12´a/b	1.53 m; 1.01 m	35.4, CH_2_
13	0.90 s	20.5, CH_3_	13´	1.05 s	31.4, CH_3_
14	1.02 s	30.1, CH_3_	14´	0.91 s	25.3, CH_3_
15	1.00 s	22.1, CH_3_	15´	0.94 s	28.2, CH_3_

**Table 3:** Inhibitory effects of sesquiterpenoids **1**–**4** on superoxide anion generation and elastase release by human neutrophils in response to *N*-Formyl-l-methionyl-l-leucyl-l-phenylalanine/ Cytochalasin B (fMLF/CB).

	Superoxide Anion		Elastase	
Compound	IC_50_ (μM) ^a^	Inh % ^b^	IC_50_ (μM) ^a^	Inh % ^b^
**1**	-	11.89 ± 5.13	-	13.69 ± 2.33 *
**2**	11.22	-	23.53	-
**3**	-	22.92 ± 4.27 *	-	35.33 ± 6.40 *
**4**	-	19.57 ± 3.69 **	7.63	-

^a^ Concentration necessary for 50% inhibition (IC_50_). ^b^ Percentage of inhibition (Inh %) at 10 μg/mL. Results are presented as means ± S.E.M (standard error of the mean) (*n* = 3). * *p* < 0.05, ** *p* < 0.01 compared with the control (solvent, dimethyl sulfoxide-DMSO).

## Supplementary Materials

The following are available online at https://www.mdpi.com/1660-3397/18/11/554/s1, Figure S1: HRESIMS spectrum of **1**; Figures S2–S7: ^1^H NMR (400 MHz), ^13^C NMR (100 MHz), HMQC, ^1^H-^1^H COSY, HMBC and NOESY Spectrum of **1** in CDCl_3_; Figure S8: HRESIMS spectrum of **2**; Figures S9–S14: ^1^H NMR (400 MHz), ^13^C NMR (100 MHz), HMQC, ^1^H-^1^H COSY, HMBC and NOESY Spectrum of **2** in CDCl_3_; Figure S15: ^1^H NMR (*S*)-MTPA ester of **3** in CDCl_3_; Figure S16: ^1^H NMR (*R*)-MTPA ester of **3** in CDCl_3_; Figure S17: HRESIMS spectrum of **4**; Figures S18–S23: ^1^H NMR (400 MHz), ^13^C NMR (100 MHz), HMQC, ^1^H-^1^H COSY, HMBC and NOESY Spectrum of **4** in CDCl_3_.
