# Supplementary material for: Novel Caryophyllane-Related Sesquiterpenoids with Anti-Inflammatory Activity from Rumphella antipathes (Linnaeus, 1758)
Source: Mar Drugs. 2020 Nov 6;18(11):554. doi: 10.3390/md18110554 (PMC7694975; doi:10.3390/md18110554)
Supplement: Supplementary file 1 [file marinedrugs-18-00554-s001.zip › marinedrugs-955994-supplementary.docx]

*Supporting Information*

Yu-Chia Chang ^1,†^, Chih-Chao Chiang ^2,†^, Yuan-Shiun Chang ^3^, Jih-Jung Chen ^4^,
Wei-Hsien Wang ^5^, Lee-Shing Fang ^5,6,7^, Hsu-Ming Chung ^8,^* and Tsong-Long Hwang ^1,9,10,11,12,^*
and Ping-Jyun Sung ^5,13,14,15,16,^*

^1^ Research Center for Chinese Herbal Medicine, Graduate Institute of Healthy Industry Technology, College of Human Ecology, Chang Gung University of Science and Technology, Taoyuan 333324, Taiwan; ycchang03@mail.cgust.edu.tw

^2^ Graduate Institute of Clinical Medical Sciences, College of Medicine, Chang Gung University, Taoyuan 333323, Taiwan; D0600501@cgu.edu.tw

^3^ Department of Chinese Pharmaceutical Sciences and Chinese Medicine Resources, College of Chinese Medicine, China Medical University, Taichung 404333, Taiwan; [yschang@mail.cmu.edu.tw](mailto:yschang@mail.cmu.edu.tw)

^4^ Faculty of Pharmacy, School of Pharmaceutical Sciences, National Yang-Ming University, 112304, Taipei, Taiwan; [chenjj@ym.edu.tw](mailto:chenjj@ym.edu.tw)

^5^ Department of Marine Biotechnology and Resources, College of Marine Sciences, National Sun Yat-sen University, Kaohsiung 804201, Taiwan; [whw@mail.nsysu.edu.tw](mailto:whw@mail.nsysu.edu.tw) (W.-H.W.); lsfang@gcloud.csu.edu.tw

(L.-S.F.)

^6^ Center for Environmental Toxin and Emerging-Contaminant Research, Cheng Shiu University, Kaohsiung 833301, Taiwan

^7^ Super Micro Mass Research and Technology Center, Cheng Shiu University, Kaohsiung 833301, Taiwan

^8^ Department of Applied Chemistry, College of Science, National Pingtung University, Pingtung 900393, Taiwan

^9^ Graduate Institute of Natural Products, College of Medicine, Chang Gung University, Taoyuan 333323, Taiwan

^10^ Department of Anesthesiology, Chang Gung Memorial Hospital, Taoyuan 333423, Taiwan

^11^ Department of Chemical Engineering, College of Environment and Resources, Ming Chi University of Technology, New Taipei City 243303, Taiwan

^12^ Research Center for Food and Cosmetic Safety, College of Human Ecology, Chang Gung University of Science and Technology, Taoyuan 333324, Taiwan

^13^ National Museum of Marine Biology and Aquarium, Pingtung 944401, Taiwan

^14^ Graduate Institute of Marine Biology, College of Marine Sciences, National Dong Hwa University, Pingtung 944401, Taiwan

^15^ Chinese Medicine Research and Development Center, China Medical University Hospital, Taichung 404394, Taiwan

^16^ Graduate Institute of Natural Products, College of Pharmacy, Kaohsiung Medical University, Kaohsiung 807378, Taiwan

***** Correspondence: shiuanmin@mail.nptu.edu.tw (H.-M.C.); [htl@mail.cgu.edu.tw](mailto:htl@mail.cgu.edu.tw) (T.-L.H.); pjsung@nmmba.gov.tw (P.-J.S.); Tel.: +886-8-766-3800 (ext. 33253); +886-3-211-8800 (T.-L.H.);
+886-8-882-5037 (P.-J.S.); Fax: +886-8-723-0305 (H.-M.C.); +886-3-211-8506 (T.-L.H.); +886-8-882-5087 (P.-J.S.)

^†^ These authors contributed equally to this work.

| Table of Contents | Page |
| --- | --- |
| **Figure S1:** HRESIMS spectrum of **1** | 2 |
| **Figure S2:** ^1^H NMR Spectrum of **1** in CDCl_3_ (400 MHz) | 2 |
| **Figure S3:** ^13^C NMR Spectrum of **1** in CDCl_3_ (100 MHz) | 3 |
| **Figure S4:** HMQC Spectrum of **1** in CDCl_3_ | 3 |
| **Figure S5:** ^1^H-^1^H COSY Spectrum of **1** in CDCl_3_ | 4 |
| **Figure S6:** HMBC Spectrum of **1** in CDCl_3_ | 4 |
| **Figure S7:** NOESY Spectrum of **1** in CDCl_3_ | 5 |
| **Figure S8:** HRESIMS spectrum of **2** | 5 |
| **Figure S9:** ^1^H NMR Spectrum of **2** in CDCl_3_ (400 MHz) | 6 |
| **Figure S10:** ^13^C NMR Spectrum of **2** in CDCl_3_ (100 MHz) | 6 |
| **Figure S11:** HMQC Spectrum of **2** in CDCl_3_ | 7 |
| **Figure S12:** ^1^H-^1^H COSY Spectrum of **2** in CDCl_3_ | 7 |
| **Figure S13:** HMBC Spectrum of **2** in CDCl_3_ | 8 |
| **Figure S14:** NOESY Spectrum of **2** in CDCl_3_ | 8 |
| **Figure S15:** ^1^H NMR (*S*)-MTPA ester of **3** in CDCl_3_ | 9 |
| **Figure S16:** ^1^H NMR (*R*)-MTPA ester of **3** in CDCl_3_ | 9 |
| **Figure S17:** HRESIMS spectrum of **4** | 10 |
| **Figure S18:** ^1^H NMR Spectrum of **4** in CDCl_3_ (400 MHz) | 10 |
| **Figure S19:** ^13^C NMR Spectrum of **4** in CDCl_3_ (100 MHz) | 11 |
| **Figure S20:** HMQC Spectrum of **4** in CDCl_3_ | 11 |
| **Figure S21:** ^1^H-^1^H COSY Spectrum of **4** in CDCl_3_ | 12 |
| **Figure S22:** HMBC Spectrum of **4** in CDCl_3_ | 12 |
| **Figure S23:** NOESY Spectrum of **4** in CDCl_3_ | 13 |

| 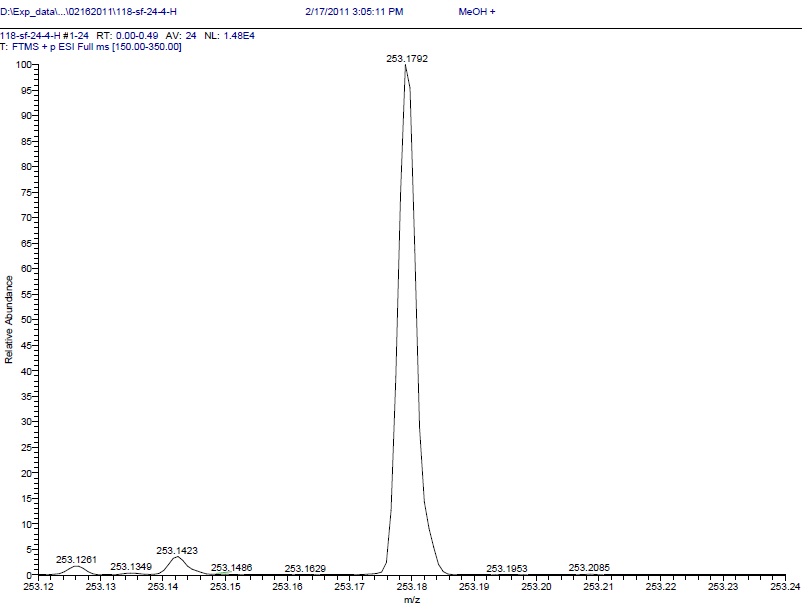 |
| --- |
| **Figure S1:** HRESIMS spectrum of **1** |
| 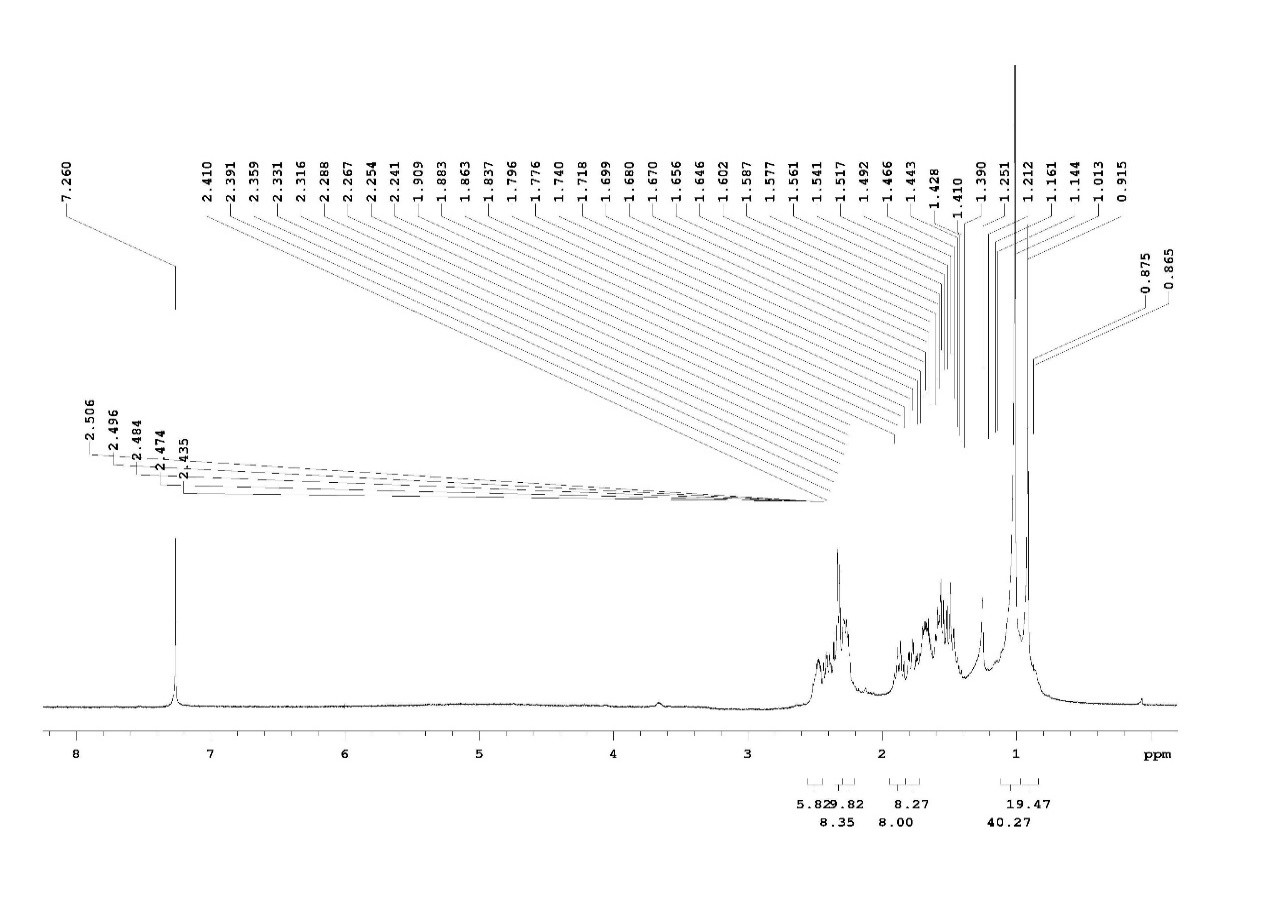 |
| **Figure S2:** ^1^H NMR Spectrum of **1** in CDCl_3_ (400 MHz) |
| 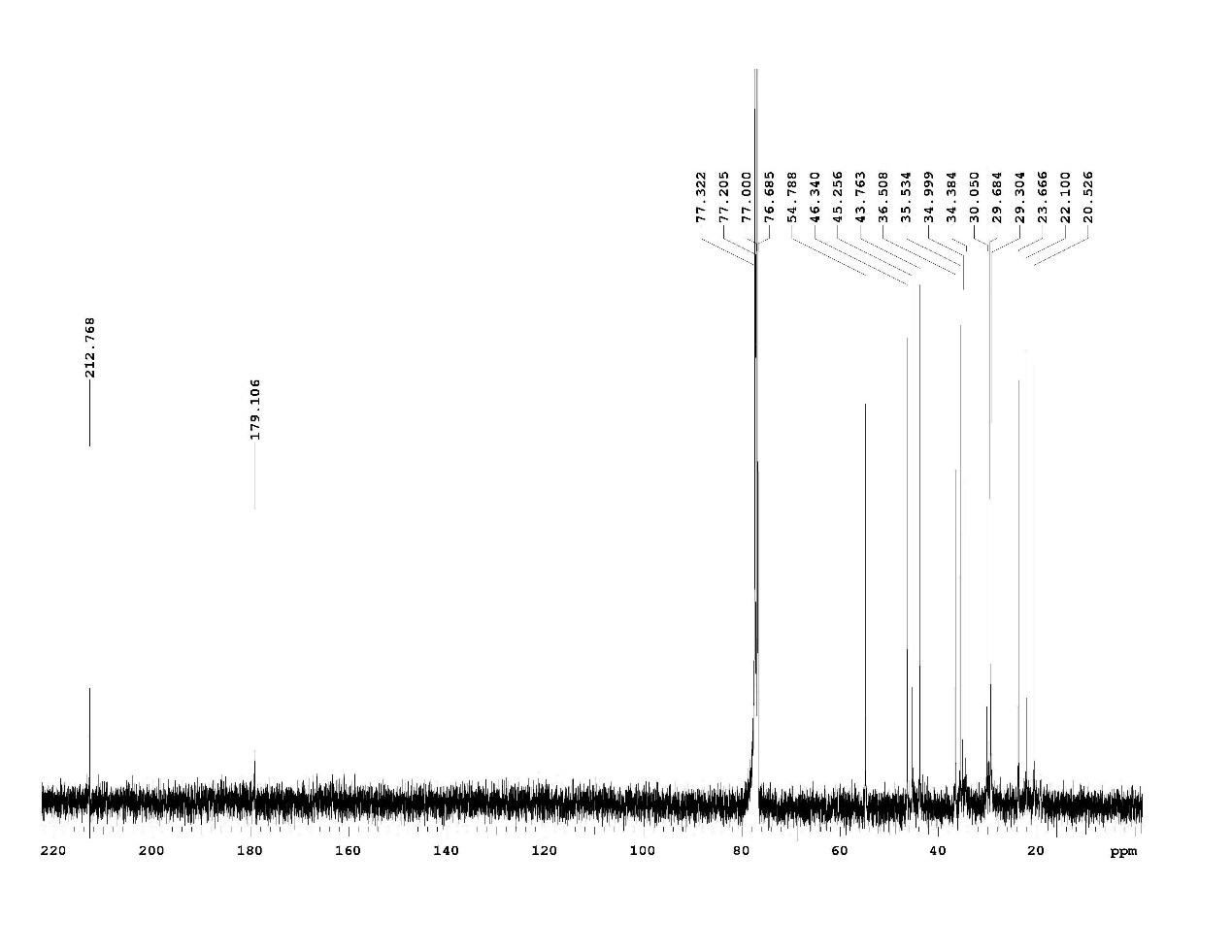 |
| **Figure S3:** ^13^C NMR Spectrum of **1** in CDCl_3_ (100 MHz) |
| 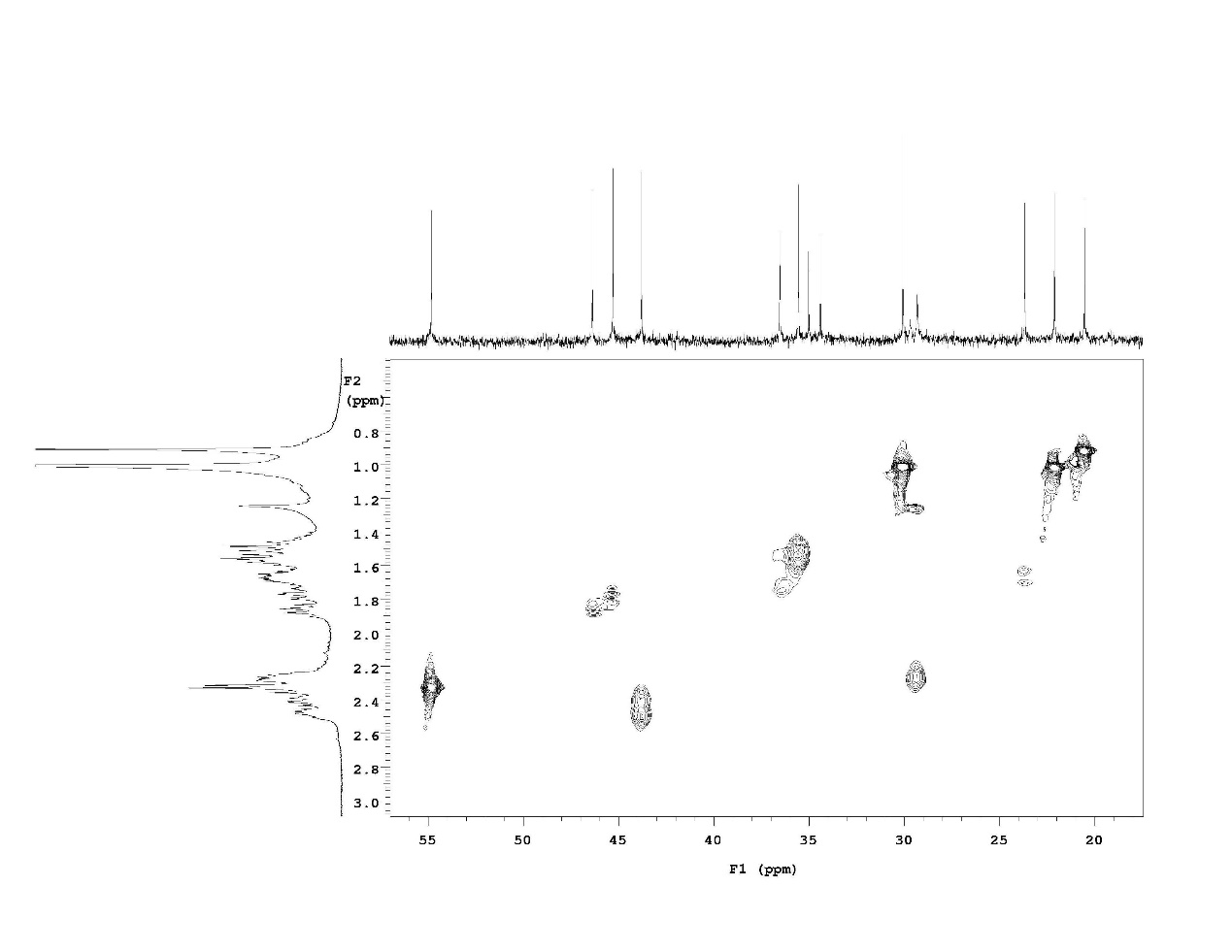 |
| **Figure S4:** HMQC Spectrum of **1** in CDCl_3_ |
| 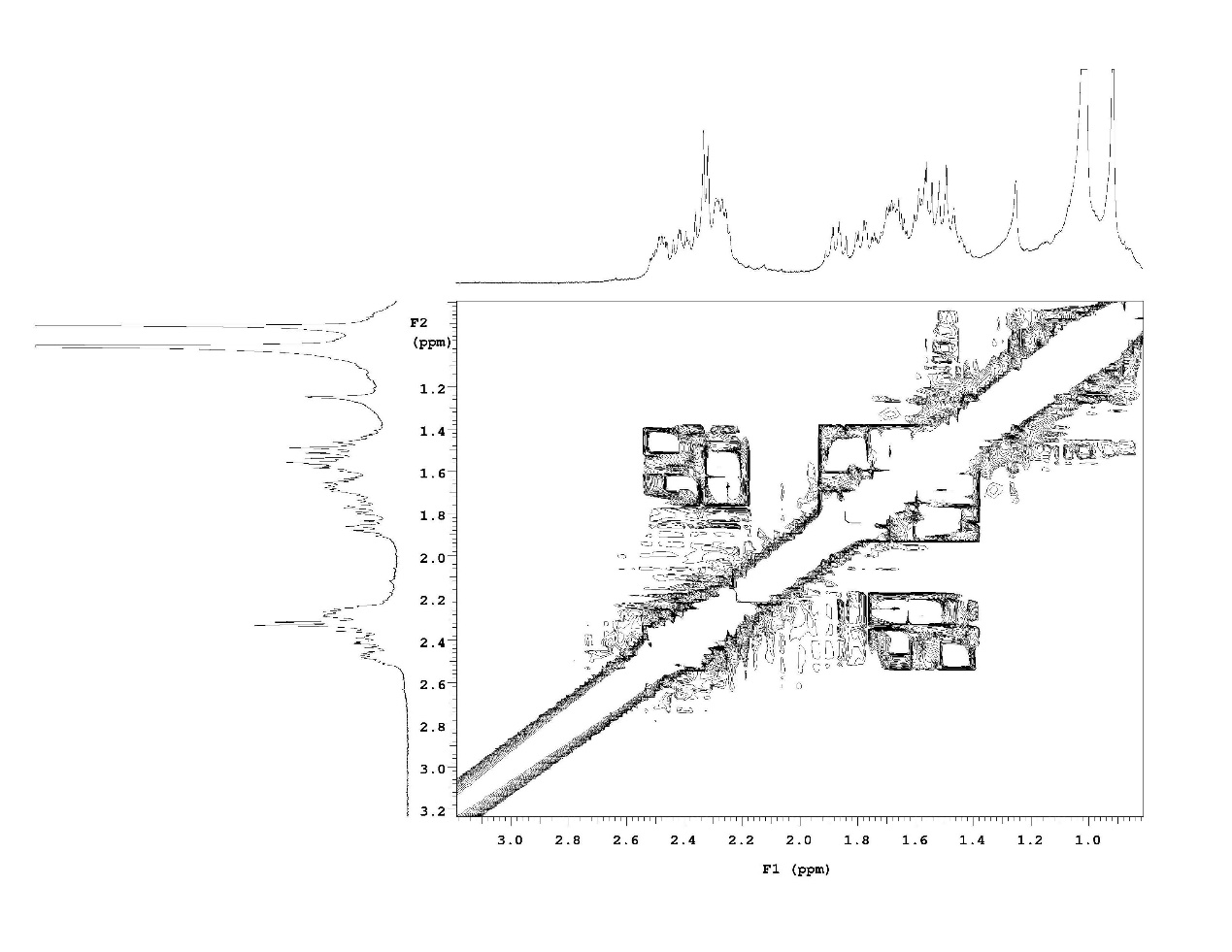 |
| **Figure S5:** ^1^H-^1^H COSY Spectrum of **1** in CDCl_3_ |
| 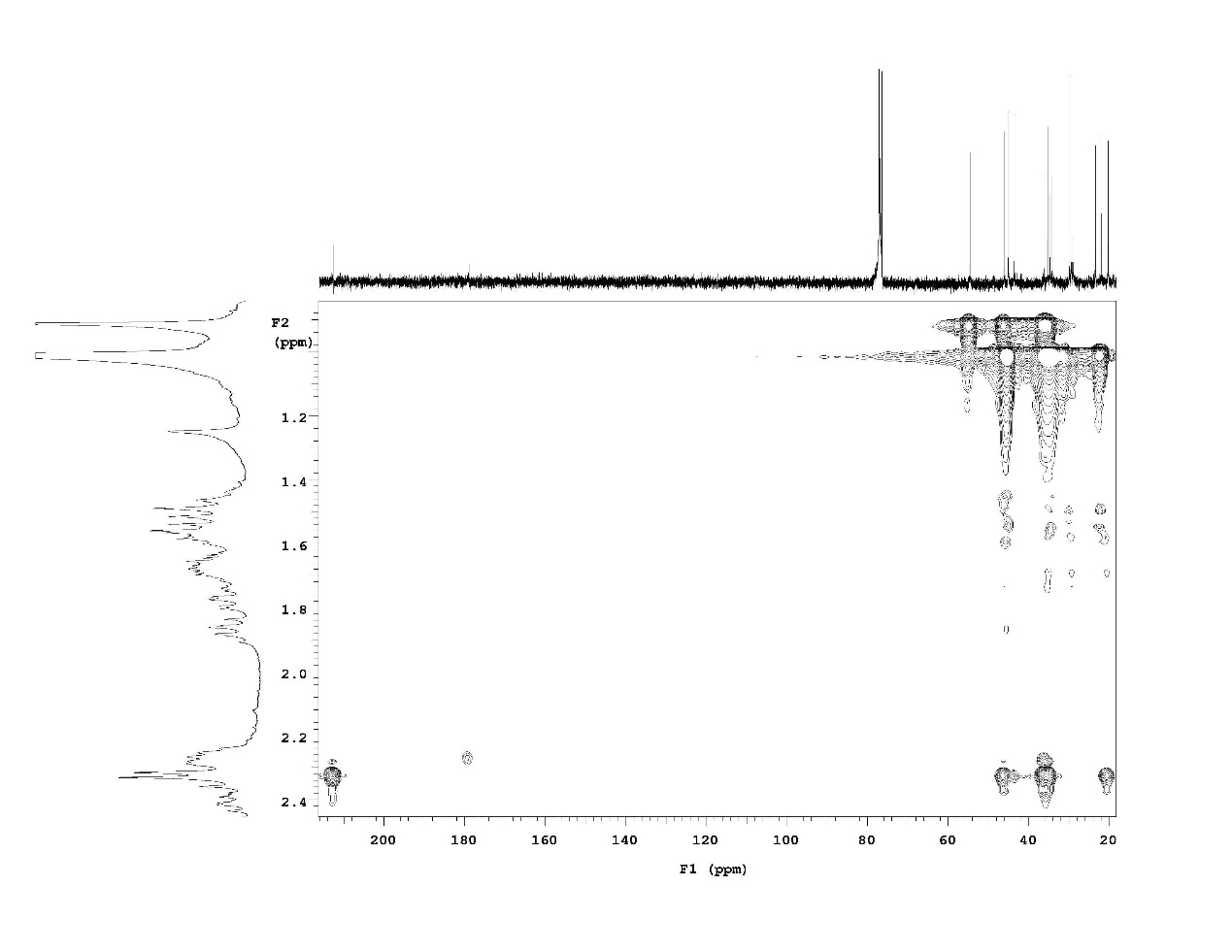 |
| **Figure S6:** HMBC Spectrum of **1** in CDCl_3_ |
| 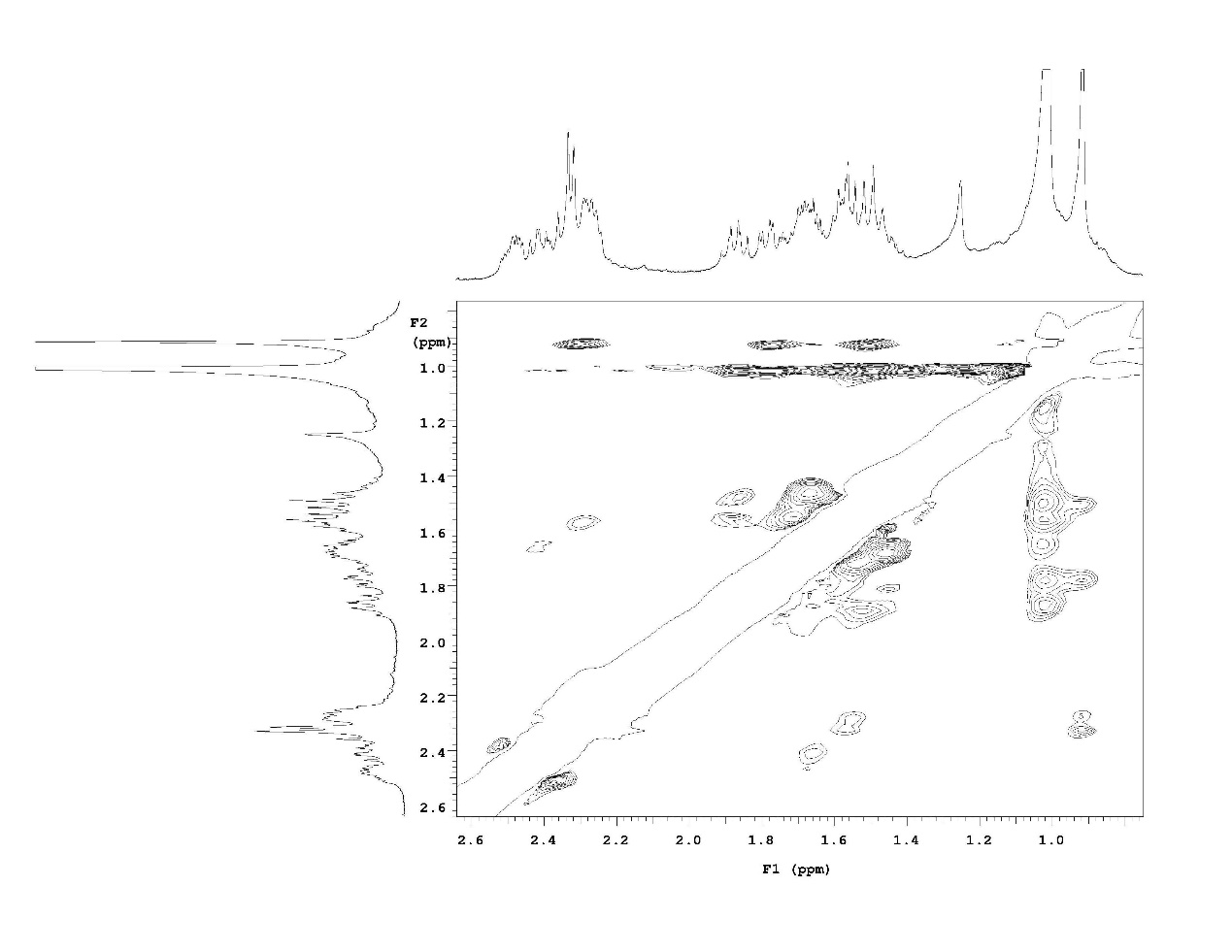 |
| **Figure S7:** NOESY Spectrum of **1** in CDCl_3_ |
| 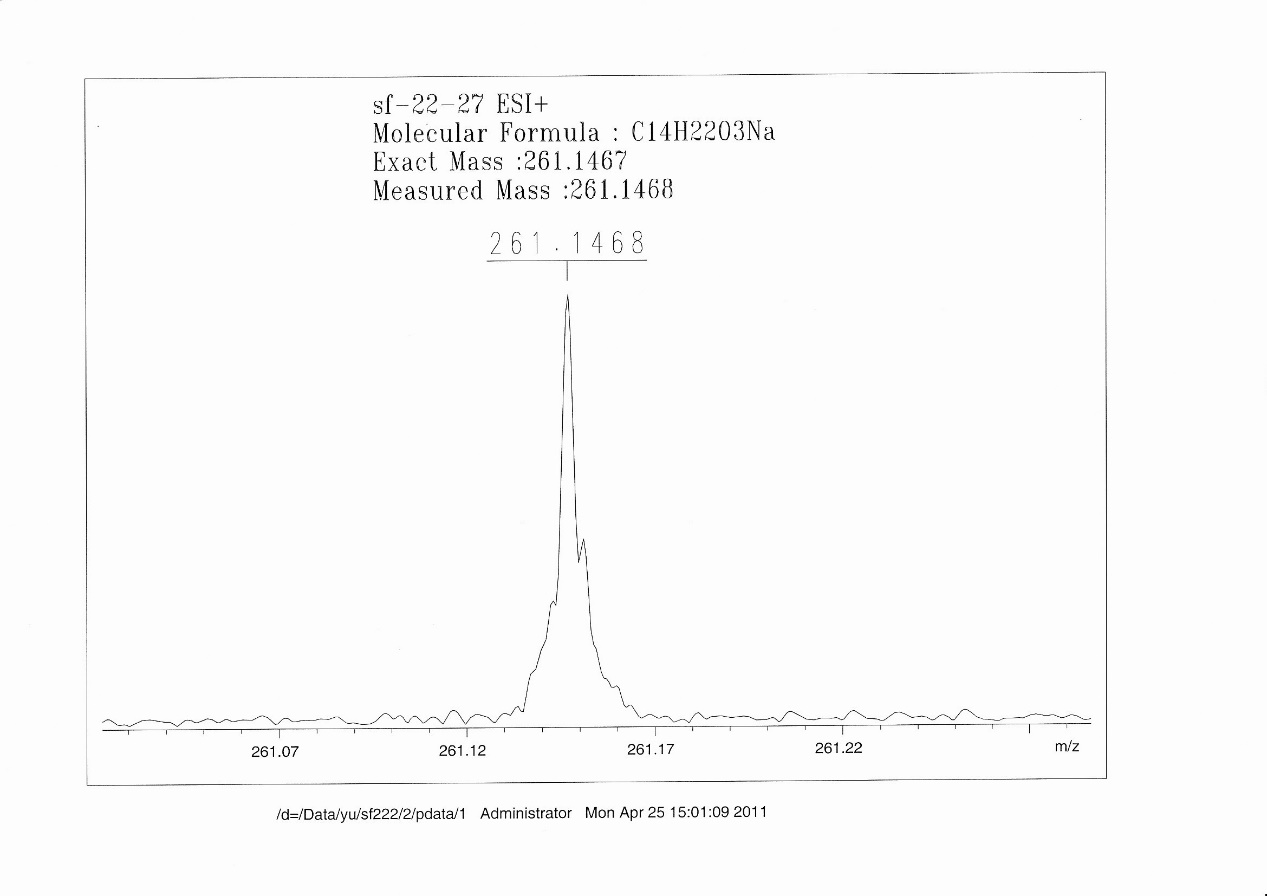 |
| **Figure S8:** HRESIMS spectrum of **2** |
| 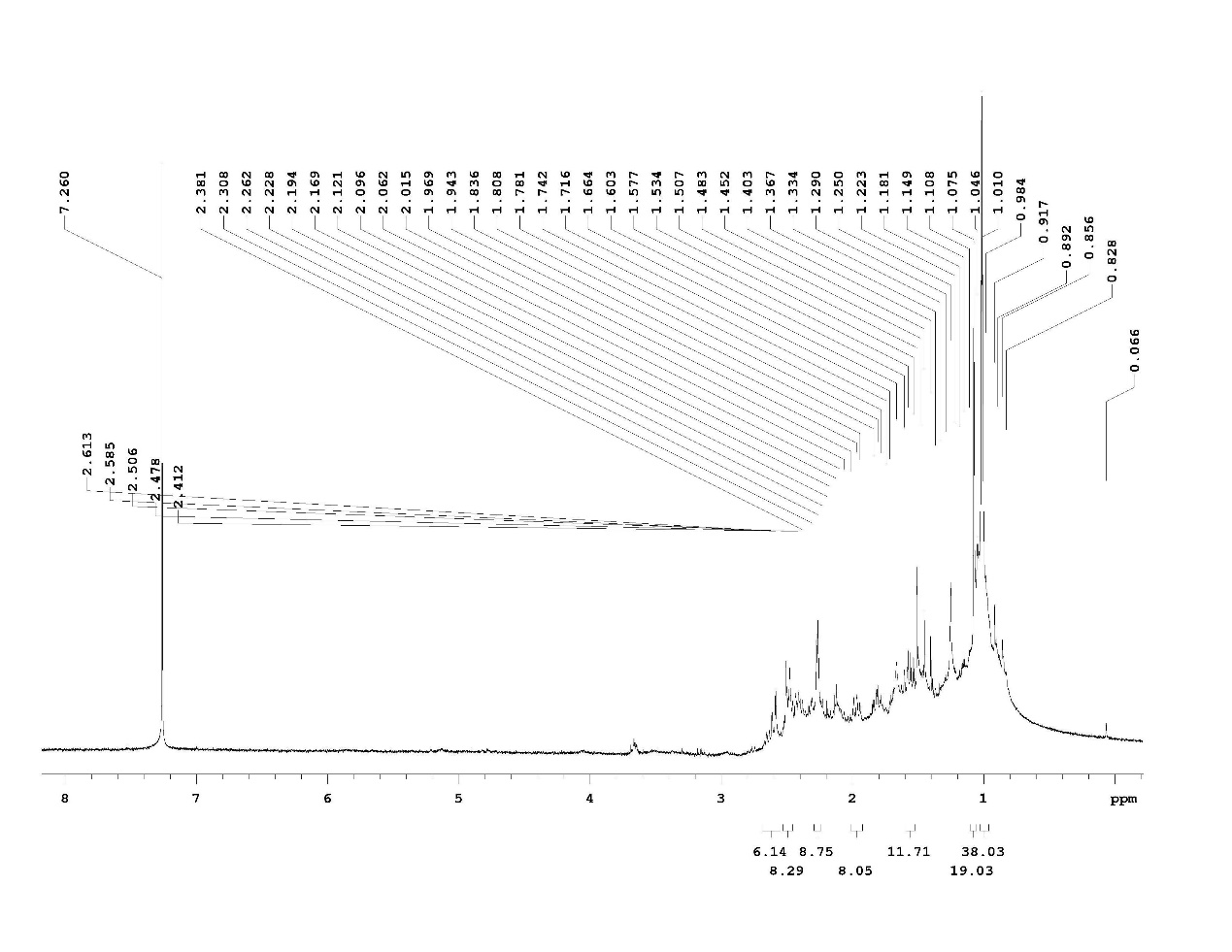 |
| **Figure S9:** ^1^H NMR Spectrum of **2** in CDCl_3_ (400 MHz) |
| 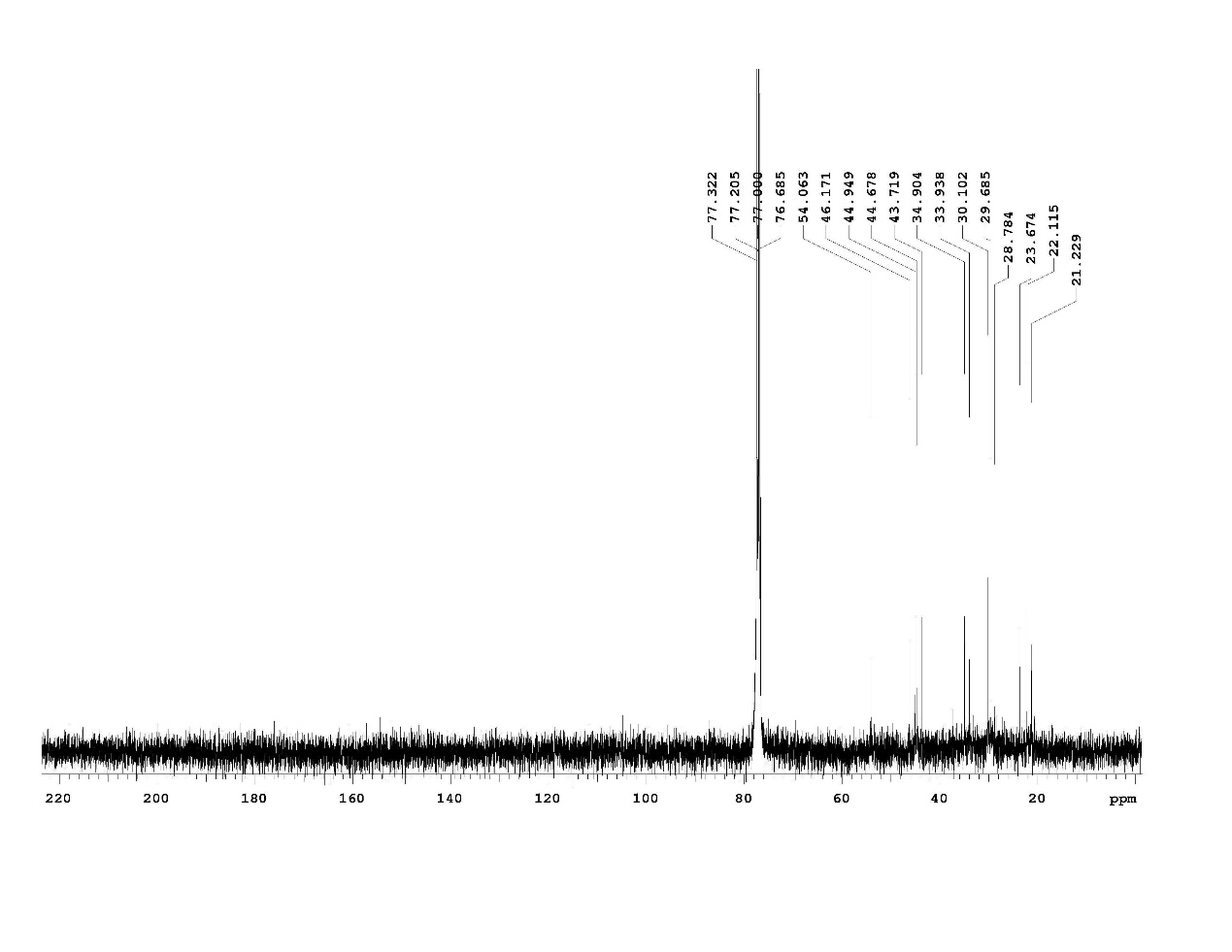 |
| **Figure S10:** ^13^C NMR Spectrum of **2** in CDCl_3_ (100 MHz) |
| 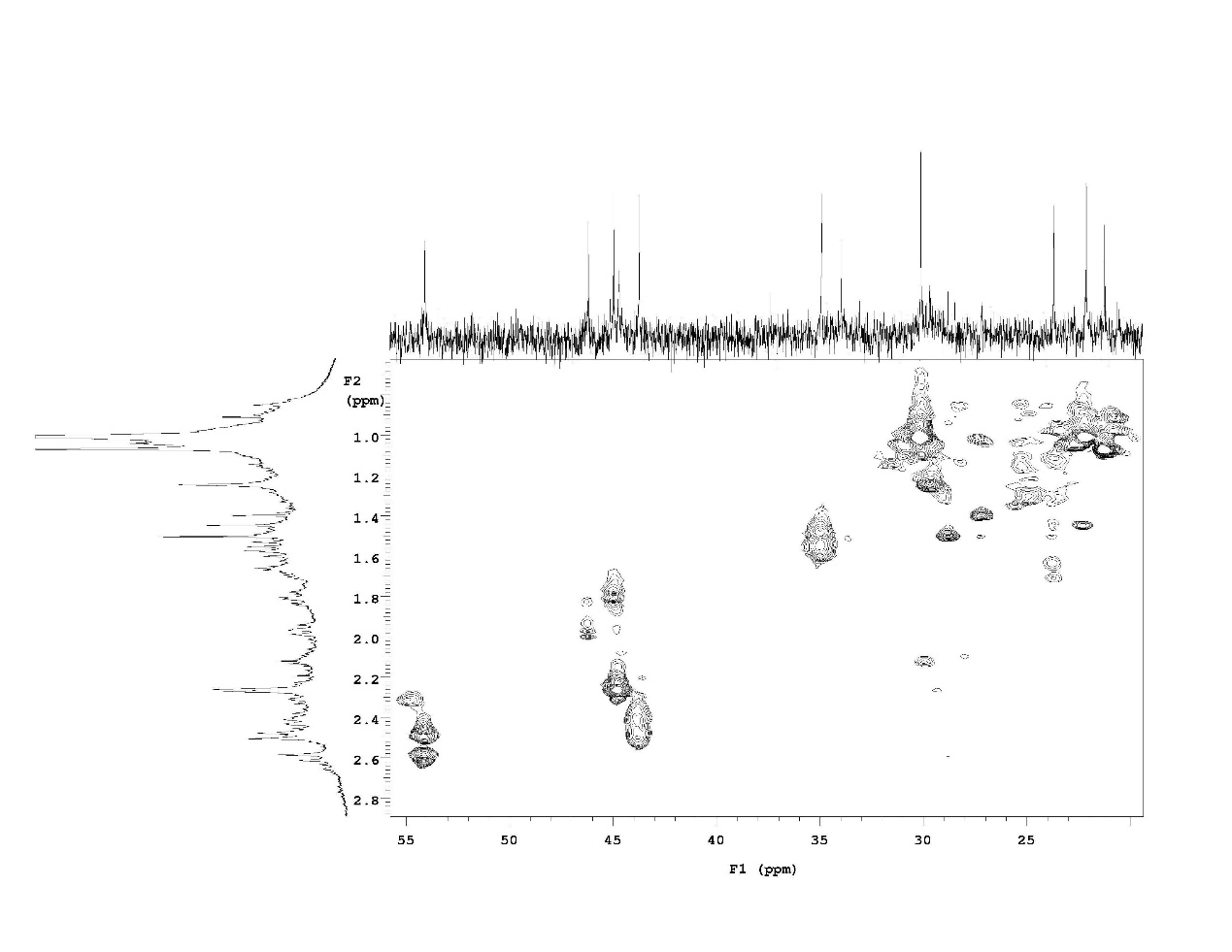 |
| **Figure S11:** HMQC Spectrum of **2** in CDCl_3_ |
| 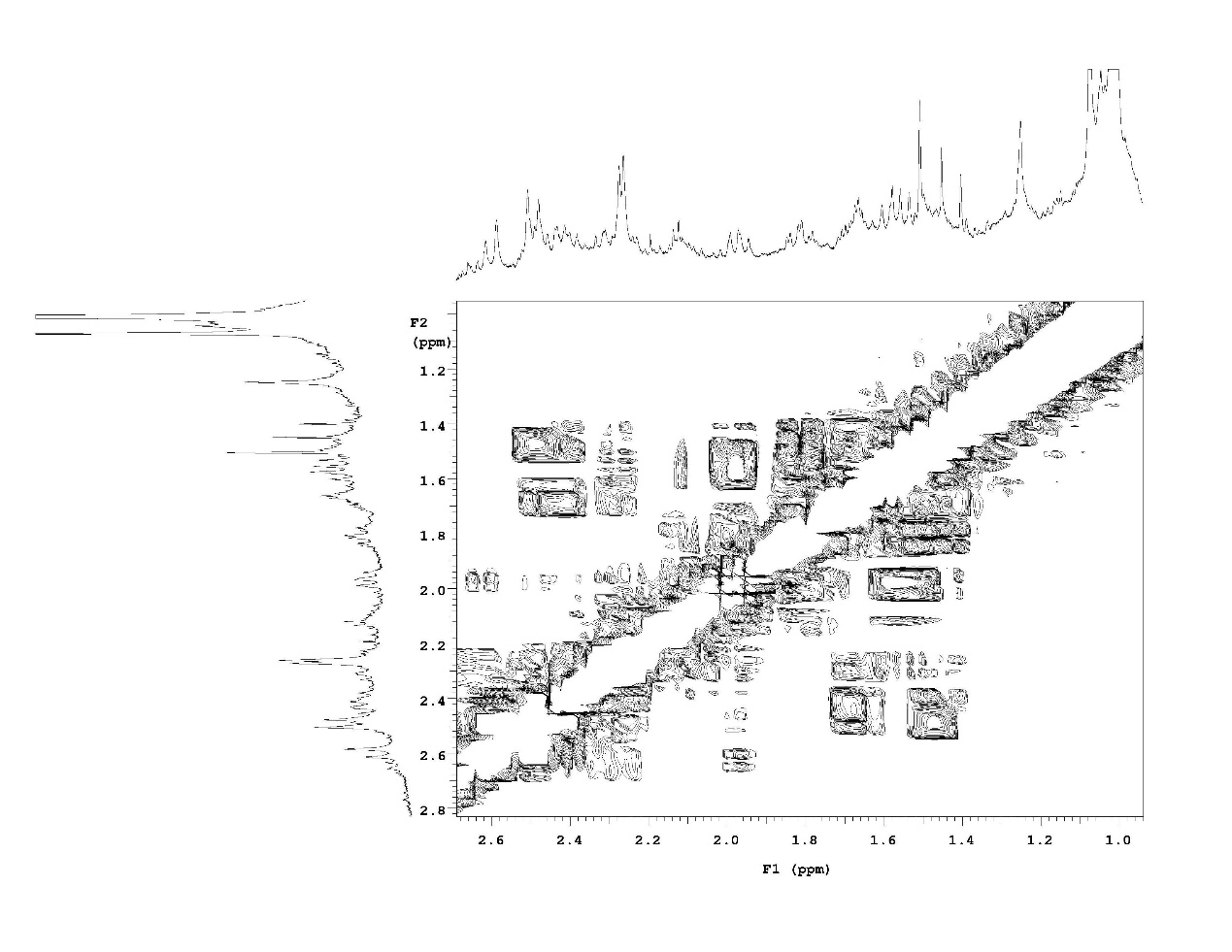 |
| **Figure S12:** ^1^H-^1^H COSY Spectrum of **2** in CDCl_3_ |
| 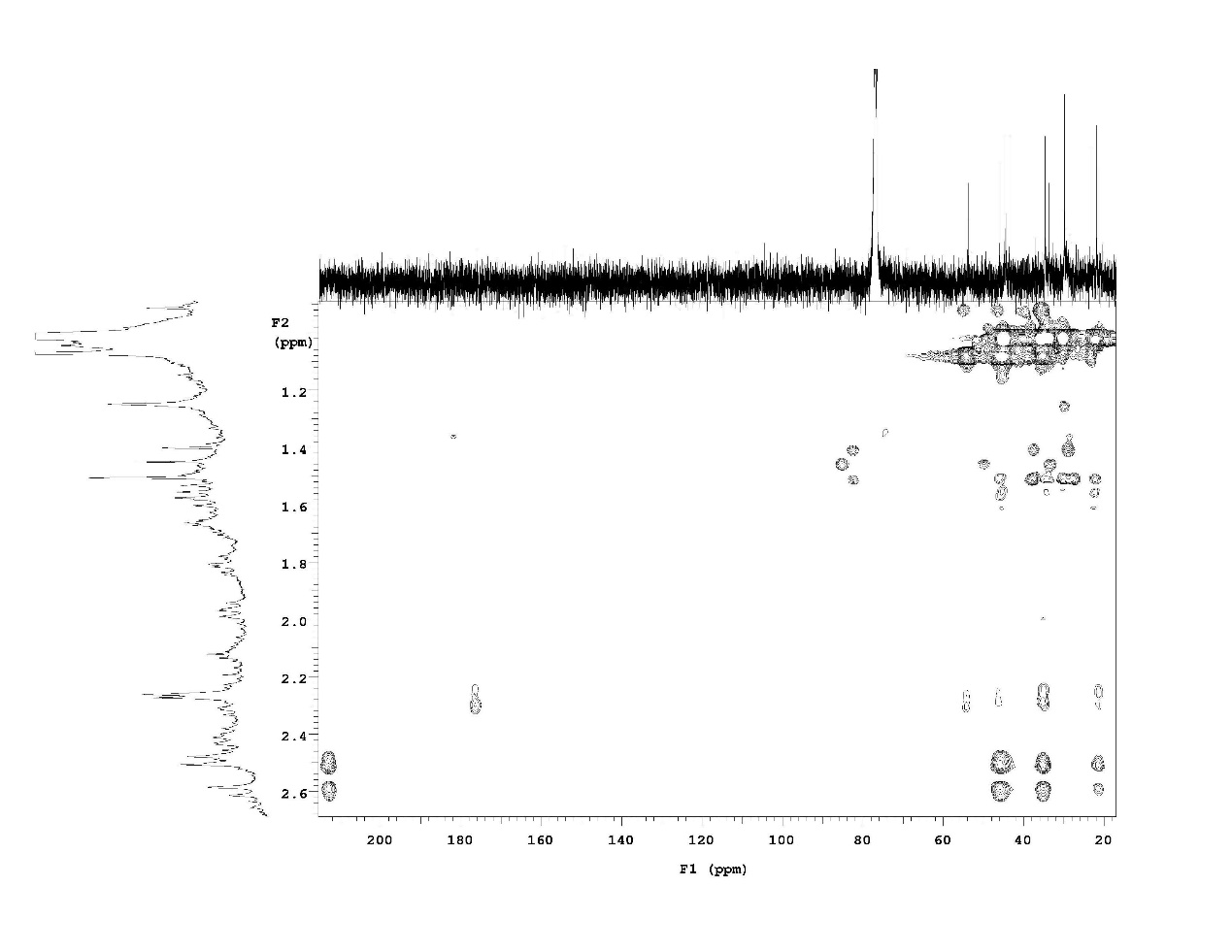 |
| **Figure S13:** HMBC Spectrum of **2** in CDCl_3_ |
| 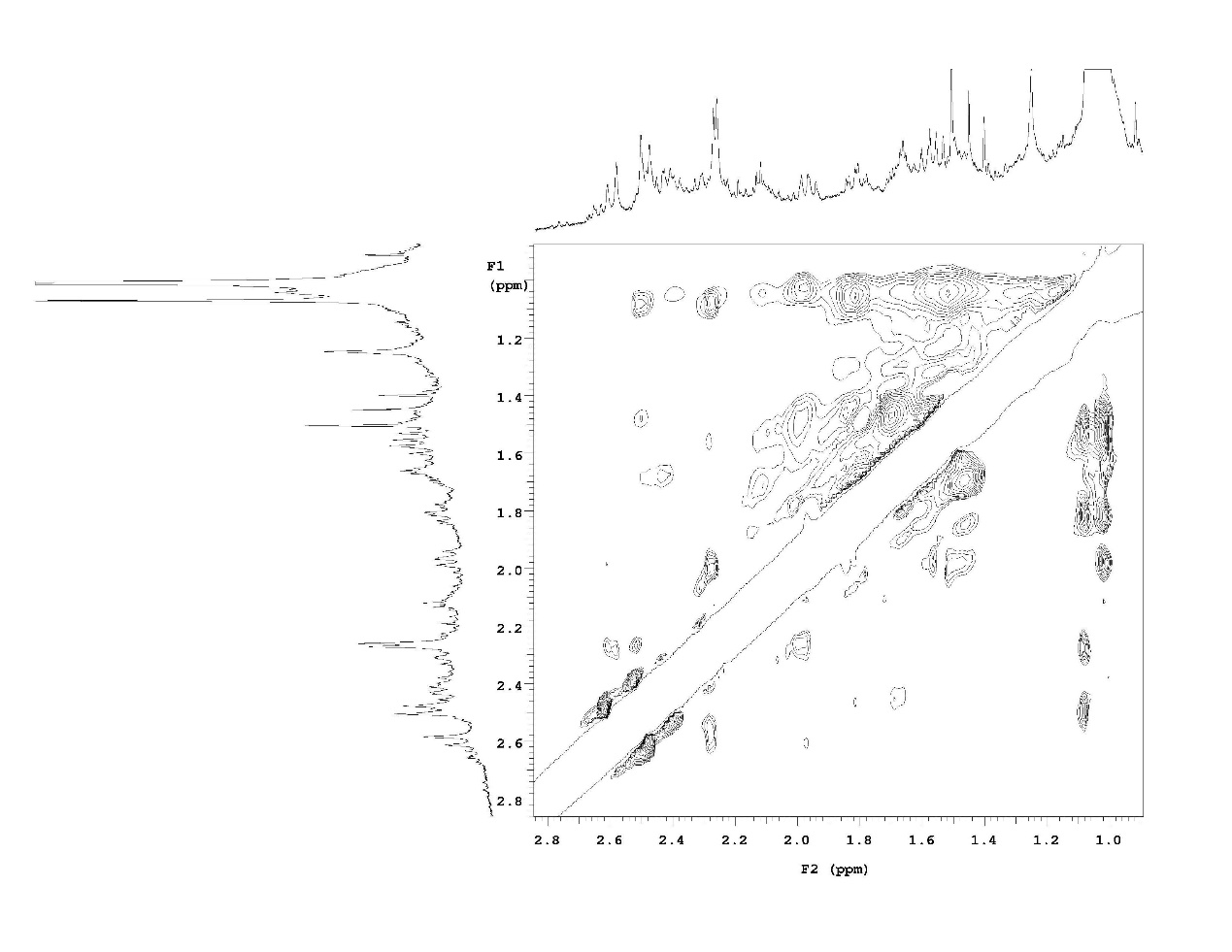 |
| **Figure S14:** NOESY Spectrum of **2** in CDCl_3_ |
| **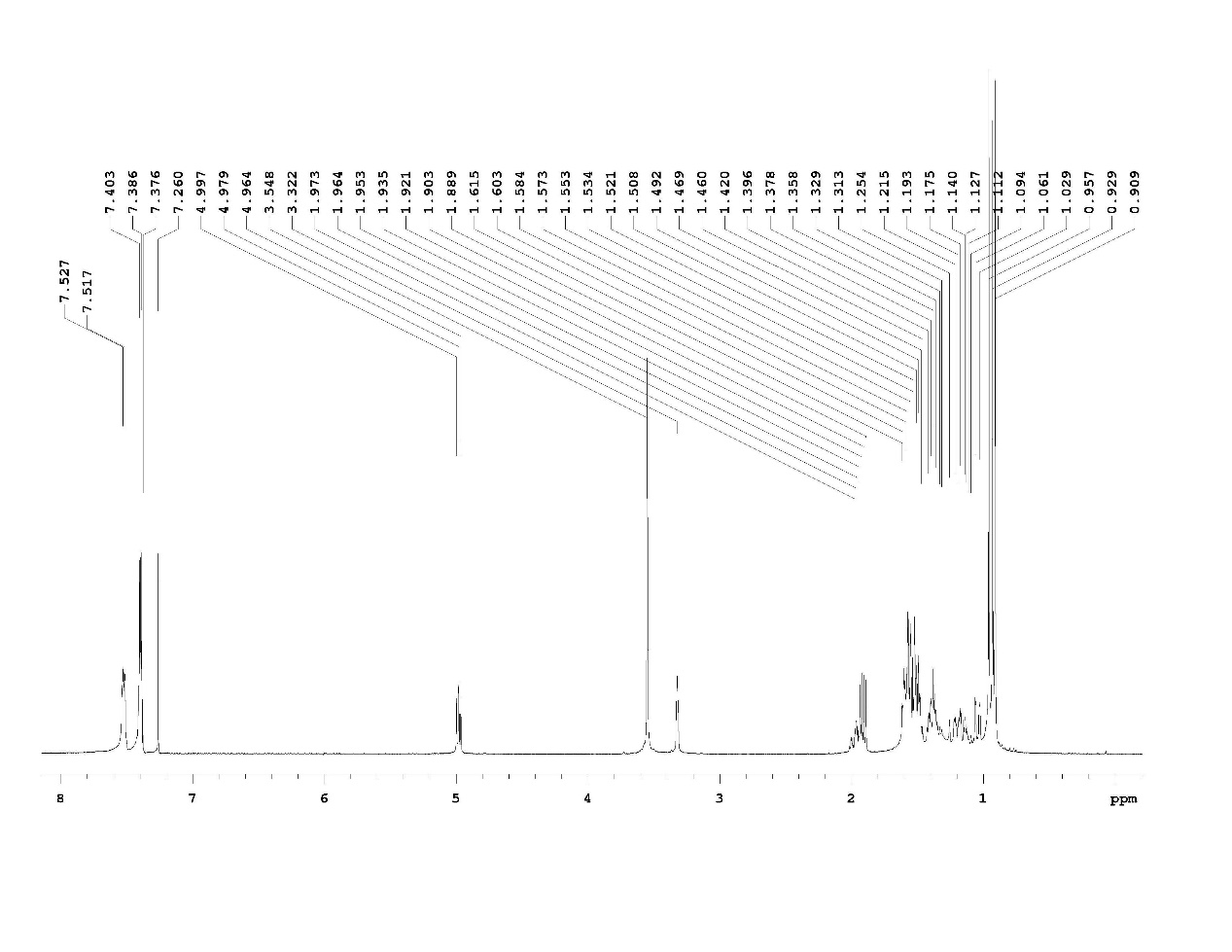** |
| **Figure S15:** ^1^H NMR (*S*)-MTPA ester of **3** in CDCl_3_ |
| **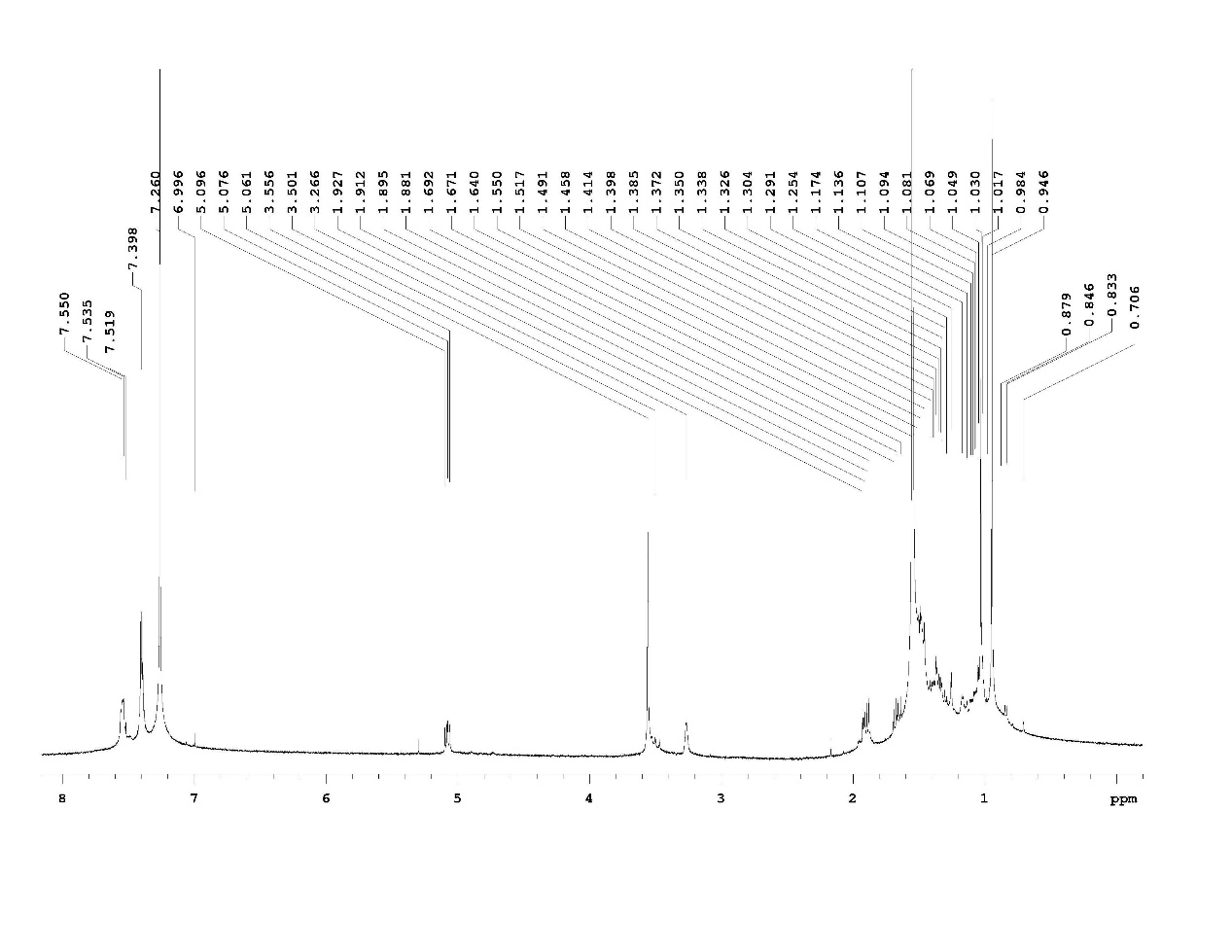** |
| **Figure S16:** ^1^H NMR (*R*)-MTPA ester of **3** in CDCl_3_ |
| 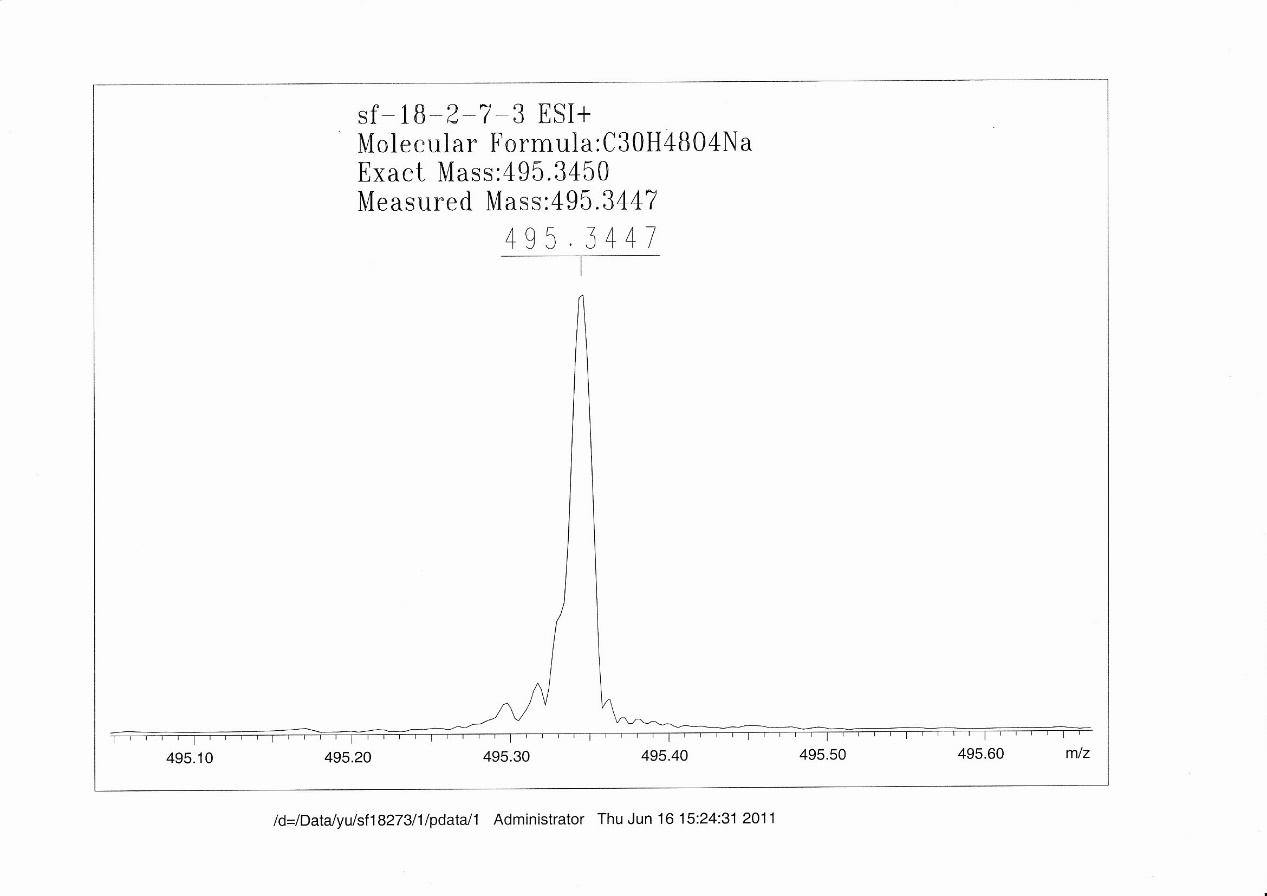 |
| **Figure S17:** HRESIMS spectrum of **4** |
| 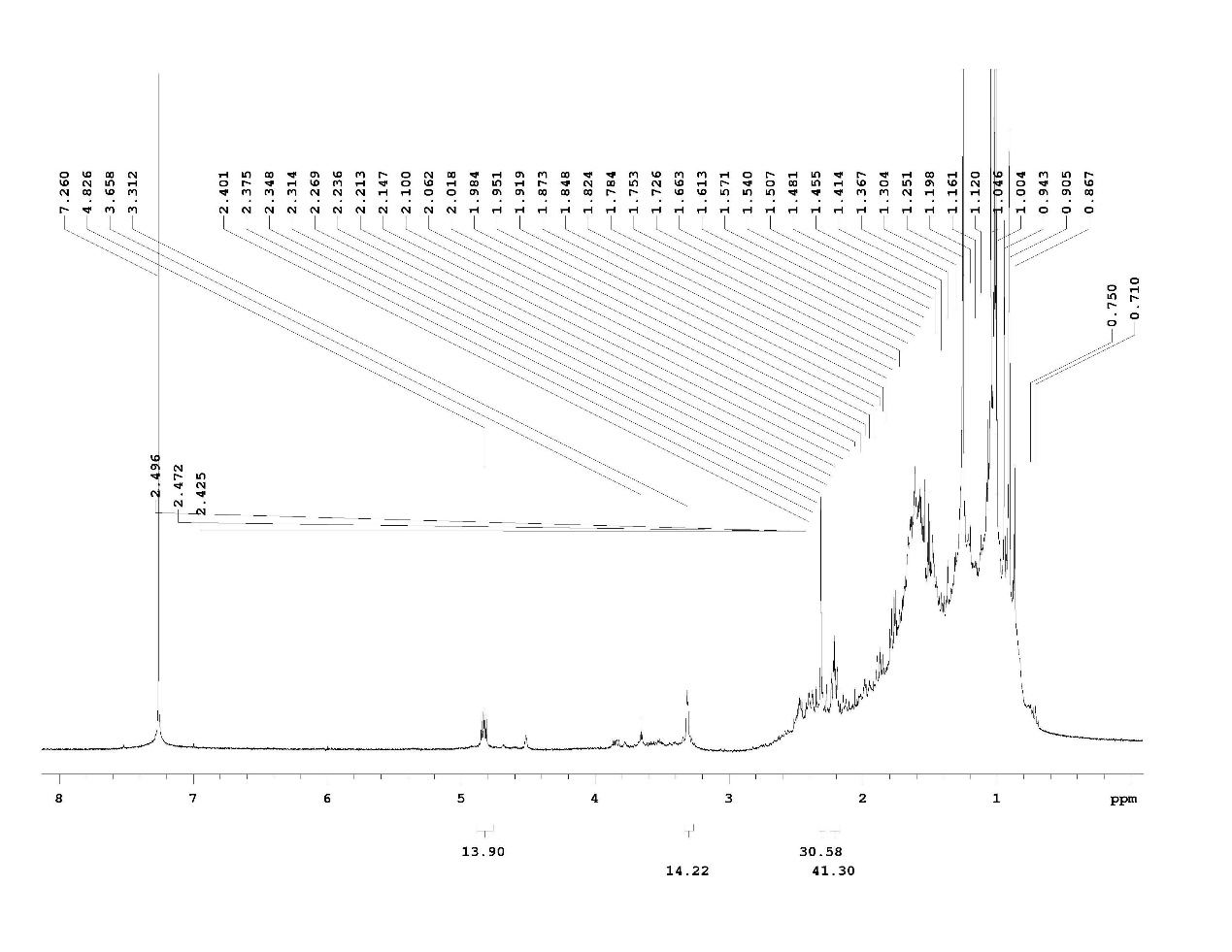 |
| **Figure S18:** ^1^H NMR Spectrum of **4** in CDCl_3_ (400 MHz) |

| 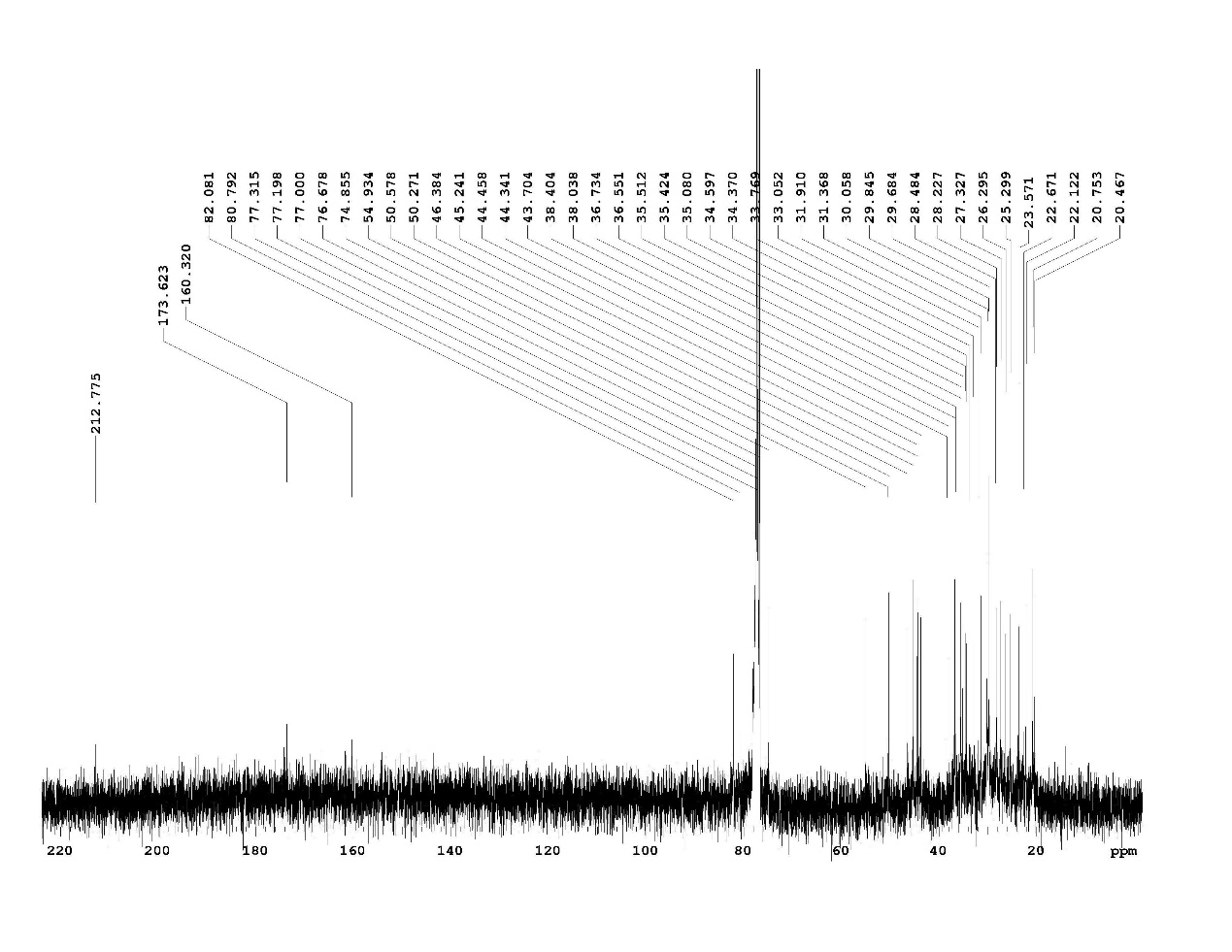 |
| --- |
| **Figure S19:** ^13^C NMR Spectrum of **4** in CDCl_3_ (100 MHz) |
| 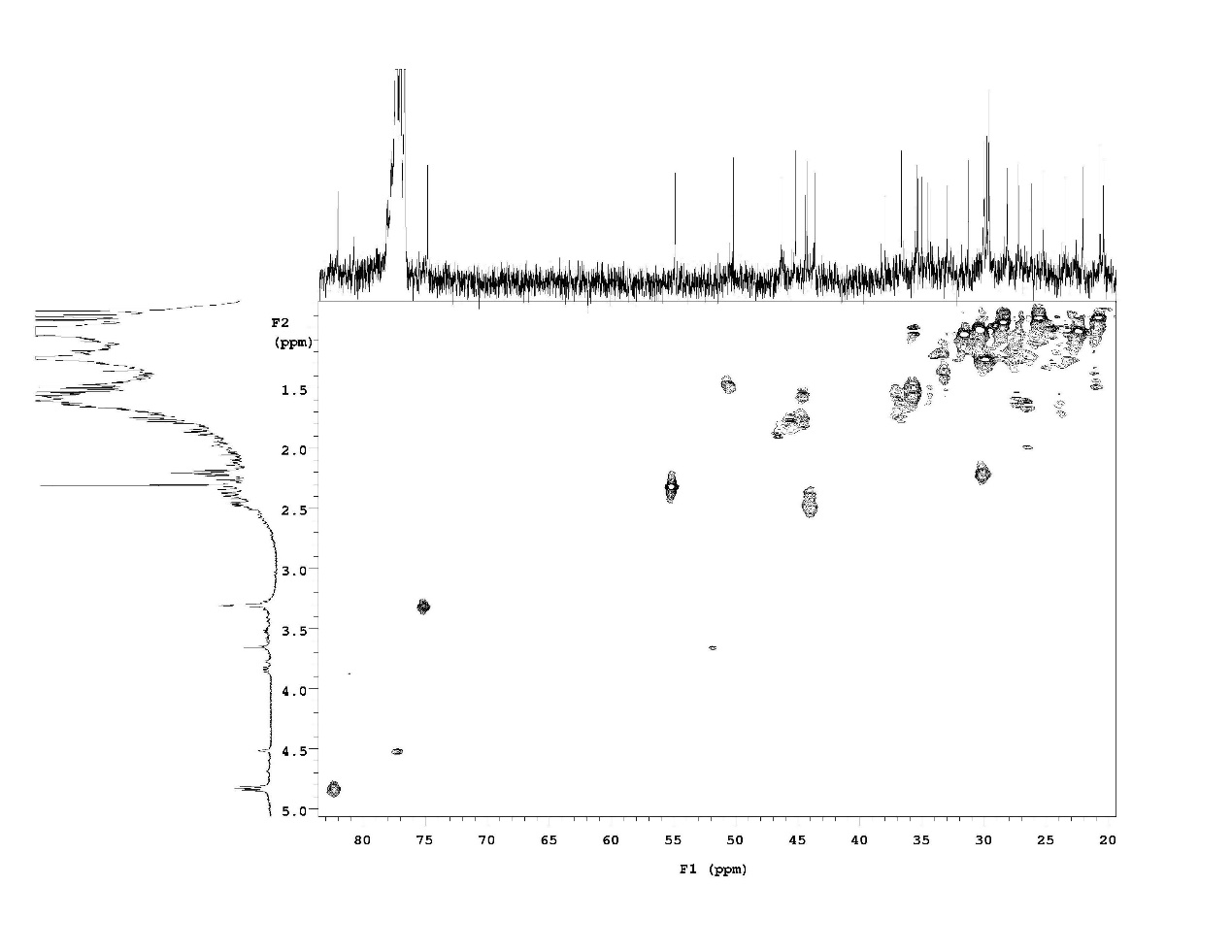 |
| **Figure S20:** HMQC Spectrum of **4** in CDCl_3_ |
| 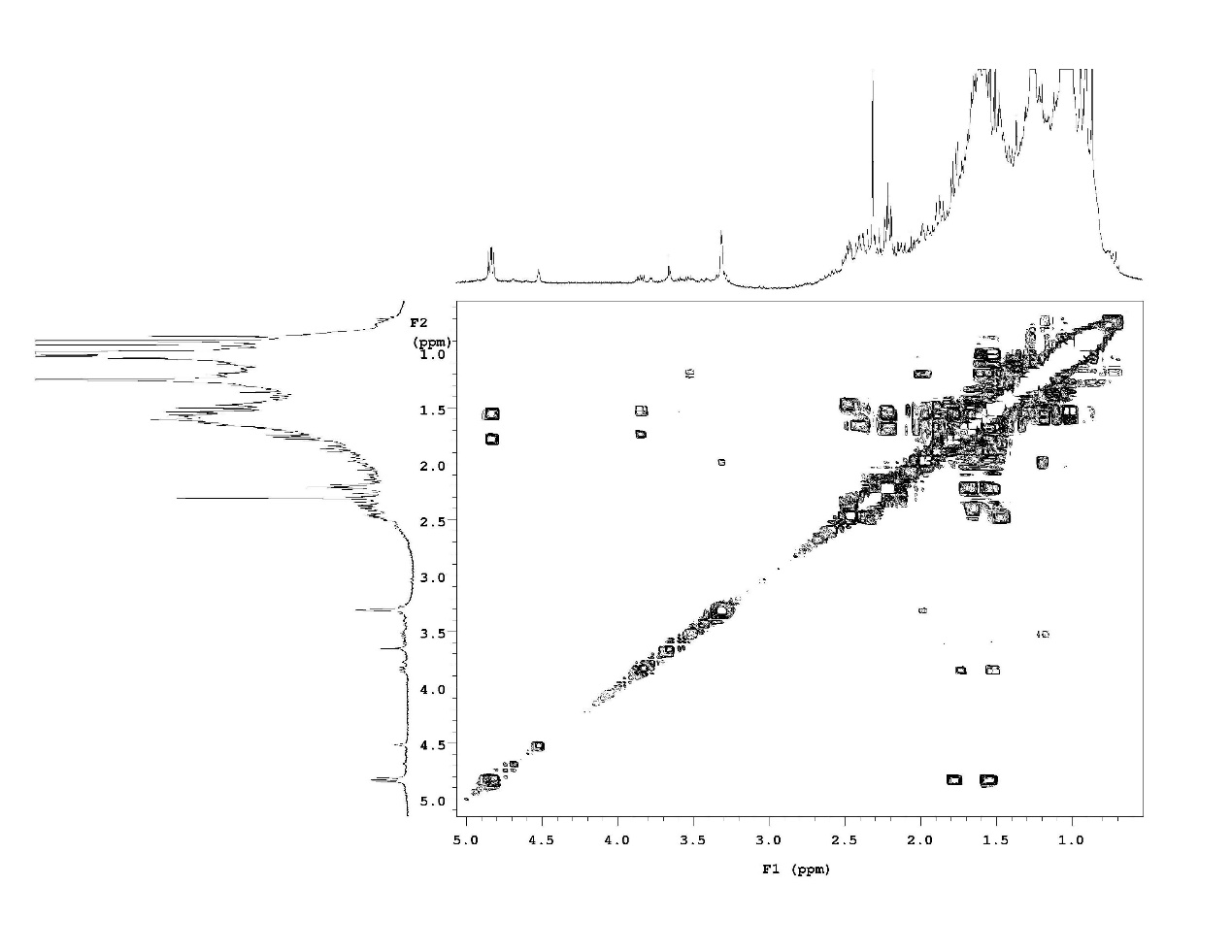 |
| **Figure S21:** ^1^H-^1^H COSY Spectrum of **4** in CDCl_3_ |
| 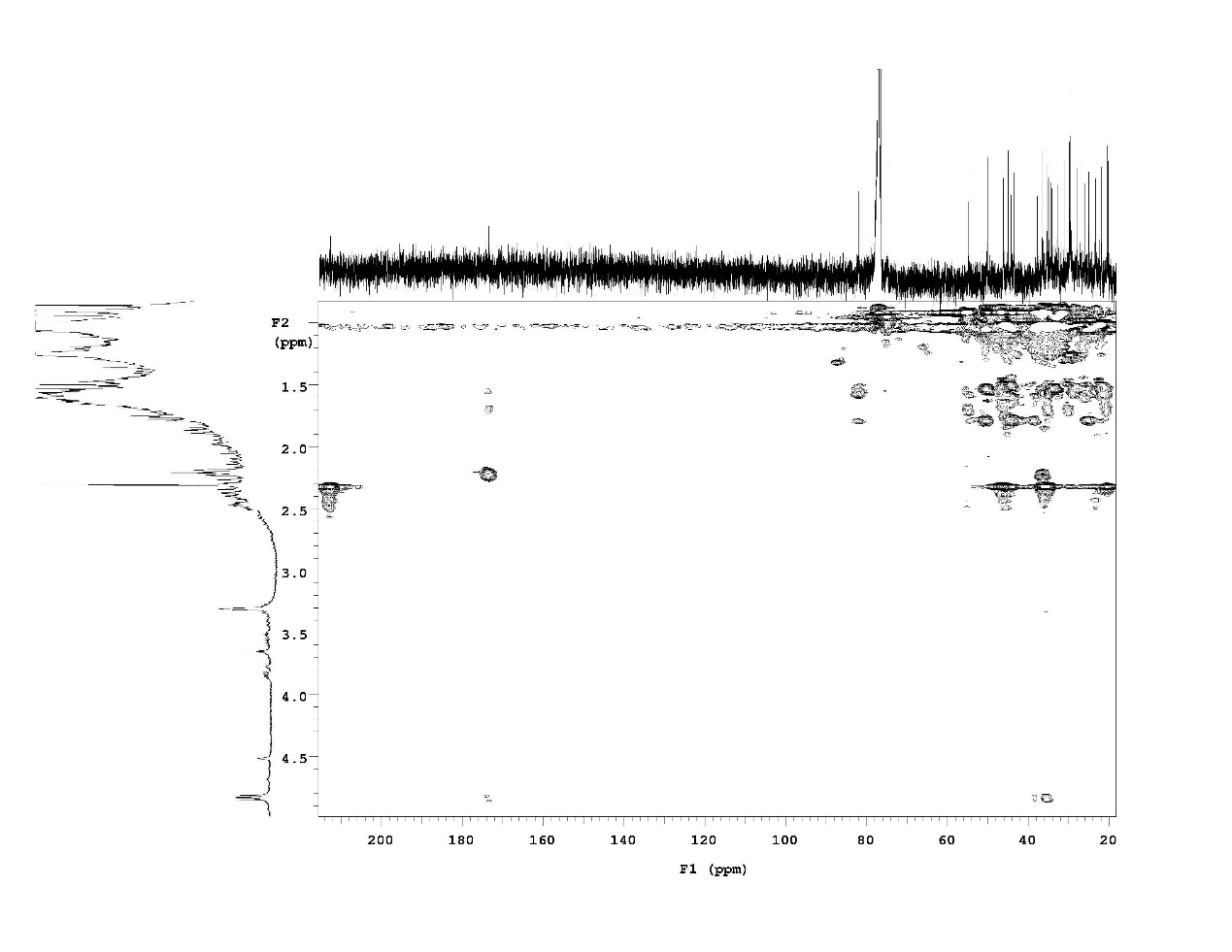 |
| **Figure S22:** HMBC Spectrum of **4** in CDCl_3_ |
| 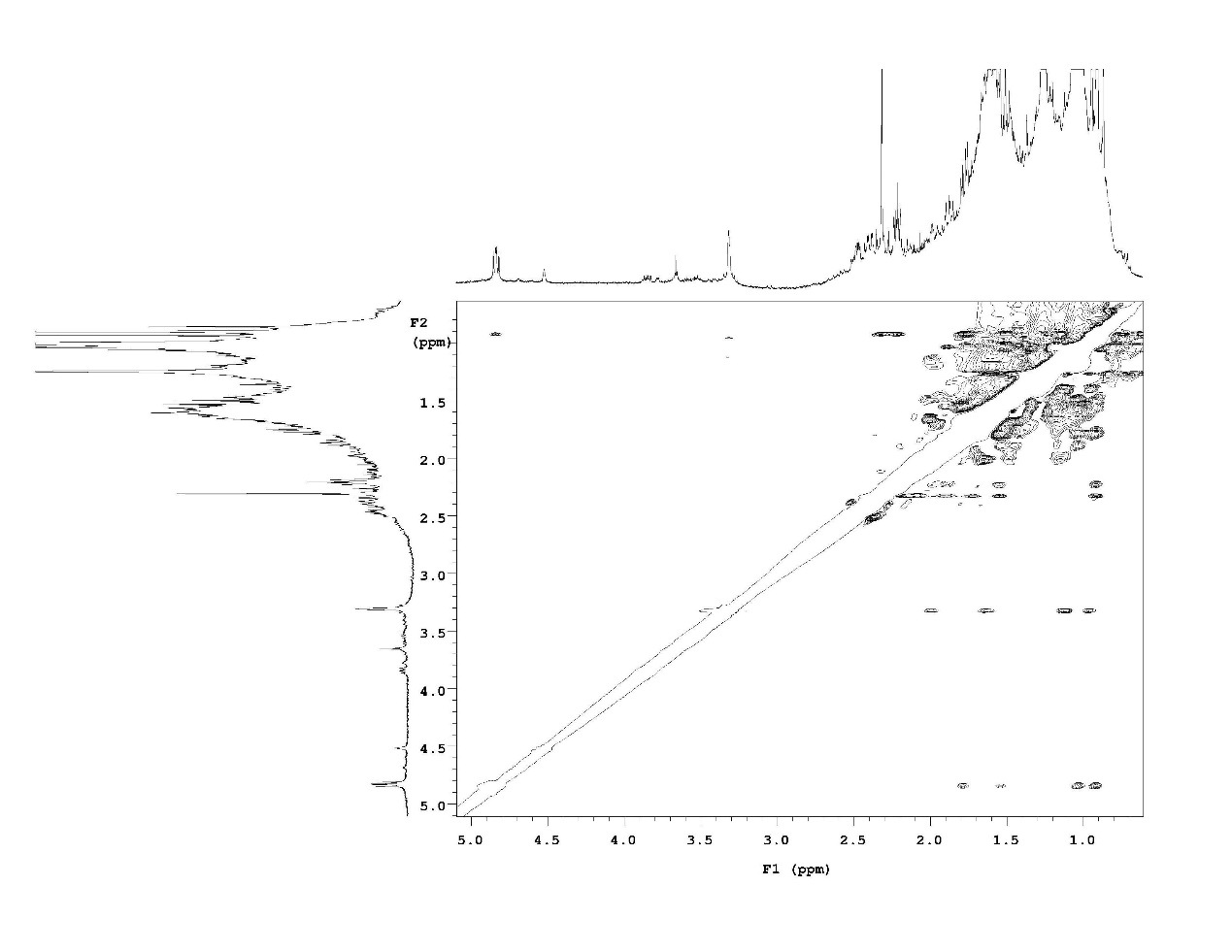 |
| **Figure S23:** NOESY Spectrum of **4** in CDCl_3_ |
